# Transcriptional regulatory network controlling the ontogeny of hematopoietic stem cells

**DOI:** 10.1101/gad.338202.120

**Published:** 2020-07-01

**Authors:** Peng Gao, Changya Chen, Elizabeth D. Howell, Yan Li, Joanna Tober, Yasin Uzun, Bing He, Long Gao, Qin Zhu, Arndt F. Siekmann, Nancy A. Speck, Kai Tan

**Affiliations:** 1 Division of Oncology, Center for Childhood Cancer Research, Children's Hospital of Philadelphia, Philadelphia, Pennsylvania 19104, USA;; 2 Department of Cell and Developmental Biology, Perelman School of Medicine, University of Pennsylvania, Philadelphia, Pennsylvania 19104, USA;; 3 Graduate Group in Cell and Molecular Biology, University of Pennsylvania, Philadelphia, Pennsylvania 19104, USA;; 4 Department of Genetics, Perelman School of Medicine, University of Pennsylvania, Philadelphia, Pennsylvania 19104, USA;; 5 Graduate Group in Genomics and Computational Biology, University of Pennsylvania, Philadelphia, Pennsylvania 19104, USA;; 6 Abramson Family Cancer Research Institute, Perelman School of Medicine, University of Pennsylvania, Philadelphia, Pennsylvania 19104, USA;; 7 Department of Pediatrics, Perelman School of Medicine, University of Pennsylvania, Philadelphia, Pennsylvania 19104, USA

**Keywords:** HSC, embryonic hematopoiesis, enhancer, epigenetics, hemogenic endothelium, regulatory network

## Abstract

Hematopoietic stem cell (HSC) ontogeny is accompanied by dynamic changes in gene regulatory networks. We performed RNA-seq and histone mark ChIP-seq to define the transcriptomes and epigenomes of cells representing key developmental stages of HSC ontogeny in mice. The five populations analyzed were embryonic day 10.5 (E10.5) endothelium and hemogenic endothelium from the major arteries, an enriched population of prehematopoietic stem cells (pre-HSCs), fetal liver HSCs, and adult bone marrow HSCs. Using epigenetic signatures, we identified enhancers for each developmental stage. Only 12% of enhancers are primed, and 78% are active, suggesting the vast majority of enhancers are established de novo without prior priming in earlier stages. We constructed developmental stage-specific transcriptional regulatory networks by linking enhancers and predicted bound transcription factors to their target promoters using a novel computational algorithm, target inference via physical connection (TIPC). TIPC predicted known transcriptional regulators for the endothelial-to-hematopoietic transition, validating our overall approach, and identified putative novel transcription factors, including the broadly expressed transcription factors SP3 and MAZ. Finally, we validated a role for SP3 and MAZ in the formation of hemogenic endothelium. Our data and computational analyses provide a useful resource for uncovering regulators of HSC formation.

## Abstract (precis)

In this study from Gao et al., the authors performed RNA-seq and histone mark ChIP-seq to define the transcriptomes and epigenomes of cells representing key developmental stages of HSC ontogeny in mice. Using a novel computational algorithm, target inference via physical connection (TIPC), they constructed developmental stage-specific transcriptional regulatory networks by linking enhancers and predicted bound transcription factors to their target promoters, thus providing a useful resource for uncovering regulators of HSC formation.

Hematopoietic stem and progenitor cells (HSPCs) differentiate from a small population of endothelial cells in the embryo called hemogenic endothelium (HE) (Slukvin and Uenishi 2019). The process of HSPC formation from HE involves an endothelial to hematopoietic transition (EHT), in which flat HE cells in a monolayer transition to rounded cells that detach from the endothelial layer and enter circulation (Slukvin and Uenishi 2019). HE is found at multiple anatomic sites in the embryo including the yolk sac and major caudal arteries (vitelline and umbilical arteries, and dorsal aorta in the aorta–gonad–mesonephros (AGM) region) (North et al. 1999). HE cells are most abundant in the major arteries at E9.5, undergo EHT between E9.5 and E12.5 (North et al. 1999; Yokomizo and Dzierzak 2010), and differentiate into HSPCs via an intermediate step in which the HSPCs briefly accumulate in clusters attached to the arterial lumen that are released into the circulation to colonize the fetal liver (FL).

Although the intra-arterial hematopoietic clusters (IACs) contain hundreds of HSPCs, only one to three of them are functional adult-repopulating HSCs (Kumaravelu et al. 2002; Yokomizo and Dzierzak 2010). IACs in mouse embryos also contain between ∼150 and ∼600 precursors of HSCs (pre-HSCs) that mature into HSCs either within or prior to colonizing the FL (Rybtsov et al. 2016; Ganuza et al. 2017). FL HSCs ultimately colonize the bone marrow (BM) where they mature into adult BM HSCs (Bowie et al. 2007). In summary, the following four populations represent a continuum of HSC ontogeny: HE > pre-HSCs > FL HSCs > BM HSCs.

Global epigenetic profiling has provided important insights into the molecules and mechanisms that regulate HSPC formation from HE. Most epigenetic studies of HSPC formation have been performed on cells generated ex vivo from mouse and human embryonic stem (ES) cells. For example, chromatin analyses combined with ChIP-seq determination of transcription factor (TF) occupancy were used to define the chromatin landscape and predict transcriptional regulatory networks (TRNs) at each step of the differentiation process from ES cells to adherent macrophages (Goode et al. 2016). This comprehensive study identified potential regulators of specific steps in the developmental trajectory that were validated in functional assays. However, ES cell cultures are thought to model yolk sac HE and HSPC formation (Vanhee et al. 2015), whereas BM HSCs are derived from the major arteries in the embryo from a distinct population of HE (Slukvin and Uenishi 2019). Little is known about the dynamics of the epigenome during HSC formation from the major arteries since many of the early precursors are extremely rare, precluding the application of most ChIP-seq protocols. There are good reasons to expect that HSPC formation in the yolk sac and major arteries will involve overlapping but distinct TRNs. It is known, for example, that HSPC formation from the major arteries requires Notch signaling and the TF MYB, whereas HSPC formation in the yolk sac requires neither (Hadland et al. 2004; Tober et al. 2008). Our previous bulk RNA-seq comparison of arterial and yolk sac HE revealed ∼2000 differentially expressed genes (DEGs) (Gao et al. 2018). We predict that distinct requirements for HSPC formation from arterial endothelium are layered on top of a canonical EHT pathway that is required in all endothelial cells.

Here we analyze the transcriptomes, and with a low cell number ChIP-seq protocol to profile the genomic locations of four histone-modification marks over the course of HSC ontogeny from HE to BM HSCs. We used a novel algorithm for predicting TRNs that we call target inference via physical connection (TIPC) to identify TFs operational at discrete stages of HSC formation, and demonstrate the importance of two broadly expressed TFs predicted by TIPC, SP3 and MAZ, in regulating the number of HE cells.

## Results

### Purification and characterization of cell populations

**Figure 1.**
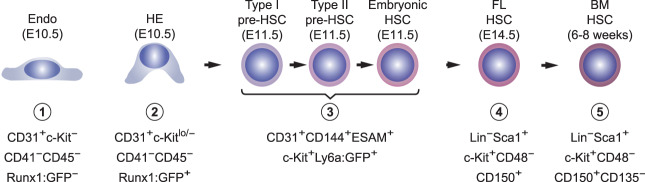
Purification of cells representing four stages of HSC ontogeny (Endo). Surface marker phenotypes of the cell populations purified. Representative sort plots are presented in Supplemental Figure S1, and functional characterization of the cells in Gao et al. (2018).

We profiled four populations of cells spanning the continuum from HE to BM-HSCs (Fig. 1). These include HE from the major arteries (dorsal aorta, umbilical, and vitelline) of E10.5 embryos, which we separated from non-HE endothelial cells (Endo) based on expression of green fluorescent protein (GFP) from the endogenous *Runx1* locus (Supplemental Fig. S1A; Lorsbach et al. 2004). We also collected GFP^−^ Endo cells for comparison. We previously showed, using the same markers, that one in 43 HE cells and one in seven Endo cells form endothelial tubes in culture (Gao et al. 2018), similar to the relative frequencies previously reported by Swiers et al. (2013), demonstrating their functional endothelial properties. On the other hand, only HE cells (one in 42) could differentiate into CD45^+^ hematopoietic cells in culture (compared with 1:20,000 Endo cells), confirming separation of functional HE and Endo (Gao et al. 2018). We also purified pre-HSCs, which cannot directly engraft adult recipients, but mature into adult-repopulating HSCs (Supplemental Fig. S1B; Ivanovs et al. 2011). All HSCs and pre-HSCs in the major arteries express a transgene from which GFP is expressed from the *Ly6a* (Sca1) regulatory sequences (de Bruijn et al. 2002; Tober et al. 2018). Only ∼15% of IAC cells are Ly6a:GFP^+^; therefore, by sorting GFP^+^ IAC cells from Ly6a:GFP transgenic mice we could enrich for pre-HSCs and HSCs. We refer to this population as pre-HSCs, because the pre-HSCs greatly outnumber the HSCs. Finally, we purified E14.5 FL HSCs and adult BM HSCs (Supplemental Fig. S1C,D). On average, we used 83,157 and 21,223 purified cells from each population for RNA-seq and ChIP-seq assays, respectively (Supplemental Tables S1, S2).

### Transcriptome dynamics during HSC ontology

**Figure 2.**
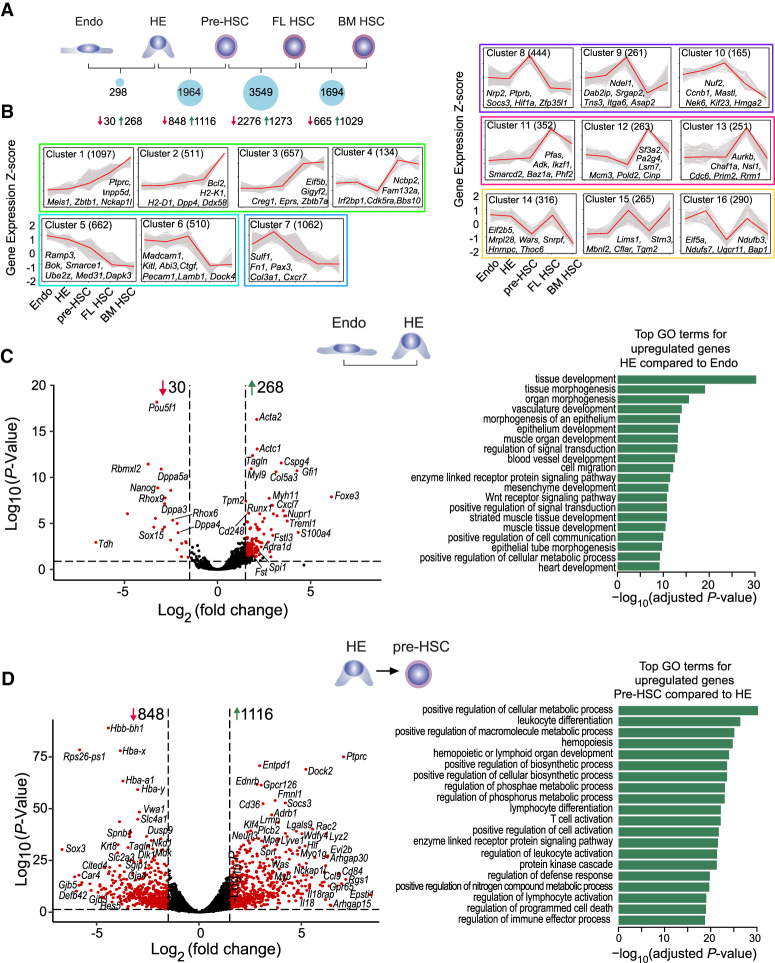
Transcriptome dynamics during HSC ontogeny. (*A*) Number of DEGs between adjacent developmental stages with corresponding down-regulated (red arrow) and up-regulated genes (green arrow) shown *below*. Some DEGs in pairwise stage comparison overlap. DEGs were identified using FDR < 0.01 and fold change ≥ 2 (ANOVA, multiple-testing corrected *P*-value < 0.01). (*B*) Sixteen expression clusters during HSC ontogeny. Expression profiles were clustered using the STEM algorithm and all 5025 DEGs among all stages. *Y*-axis values are gene-wise *Z*-score of FPKM values. *X-*axis and *Y*-axis values are indicated in the *bottom left* graph. For each cluster, the numbers of genes are shown in parentheses. The 16 clusters are further organized into six color-coded groups based on their overall expression profiles. Group 1 (clusters 1–4) in are boxed in green, group 2 (clusters 5–6) in cyan, group 3 (cluster 7) in blue, group 4 (clusters 8–10) in purple, group 5 (clusters 11–13) in magenta, and group 6 (clusters 14–16) in yellow. Example genes for each cluster are also shown. (*C*, *left*) Volcano plot of DEGs between Endo and HE stage; selected genes are listed. (*Right*) The top GO terms for genes up-regulated in HE relative to Endo. (*D*) Same as in *C*, for differences between HE and pre-HSC stages.

To identify changes in transcriptomes during HSC ontogeny, we performed RNA-seq using biological replicates of sorted cells at four developmental stages (HE, pre-HSC, FL HSC, and BM HSC) plus Endo (Supplemental Fig. S2). We detected an average of 12,511 expressed genes at a FPKM threshold of one in each population, and 5025 differentially expressed genes between two adjacent developmental stages (Fig. 2A; Supplemental Table S3). Using the short-time series expression miner (STEM) algorithm (Ernst et al. 2005), we identified sixteen expression clusters among the 5025 genes with greater than or equal to twofold changes between two adjacent developmental stages (Fig. 2B). The expression clusters are further categorized into six groups based on their expression dynamics across developmental stages. Group 1 genes (clusters 1–4) gradually increase in expression over HSC ontogeny, with peak levels in FL and/or BM HSCs, and are enriched for Gene Ontology (GO) terms associated with HSCs (Supplemental Fig. S3A). Group 2 genes (clusters 5–6) are enriched for endothelial cell migration and motility. Genes that peak in HE (group 3; cluster 7) are enriched for inflammatory genes. Genes that peak in pre-HSCs (group 4; clusters 8–10) are enriched for inflammatory response and regulation of cell cycle. Genes that peak in FL HSCs (group 5; clusters 11–13) are enriched for functional HSC terms. Group 6 (clusters 14–16) have oscillating expression suggesting that some genes are only required for specific transitions (Fig. 2B; Supplemental Fig. S3A).

The smallest difference in gene expression changes (298 DEGs) occurs between Endo and HE, reflecting the recent endothelial origin of HE (Fig. 2A). Interestingly, most DEGs are up-regulated genes that are likely involved in the morphological changes necessary for EHT. These include actin and actin-related genes, *Acta2*, *Actc1*, and *Tagln*, some of which have been observed by others during EHT (Fig. 2C; Vargel et al. 2016). Hematopoietic genes, *Runx1*, *Gfi1*, and *Spi1*, which are known to play a role in EHT are also up-regulated. GO analysis of genes up-regulated in HE relative to Endo confirmed EHT-related processes such as cell migration and tissue morphogenesis (Fig. 2C). Top down-regulated genes between Endo and HE include developmental factors (*Pou5f1*, *Dppa5a*, *Dppa3*, etc.) and various metabolic and biosynthetic processes (Supplemental Fig. S3B), which is consistent with metabolic quiescence of HE (Oatley et al. 2020).

The transition from HE to pre-HSC is accompanied by much larger changes in gene expression (1116 up-regulated genes and 848 down-regulated genes). The majority of up-regulated genes are known hematopoietic genes such as *Ptprc* (CD45)*, Dock2* (Oatley et al. 2020)*, Spn* (CD43)*, Myb,* and *Neurl3*, which was recently shown to selectively mark HSC-primed HE (Fig. 2D; Hou et al. 2020). GO terms of up-regulated genes in pre-HSCs relative to HE such as leukocyte differentiation, hemopoiesis, hemopoietic or lymphoid organ development confirm fulfillment of a hematopoietic fate. The top GO terms for down-regulated genes suggest commitment to the hematopoietic fate through elimination of other cell fates (e.g., neuronal and epithelial) (Supplemental Fig. S3C).

The largest transcriptome change occurs between pre-HSCs and FL HSCs, which differentially express 3549 genes (Supplemental Fig. S3D). Up-regulated genes are enriched for hemopoiesis and other hematopoietic and lymphoid-specific terms, while down-regulated genes appear to show further commitment to the hematopoietic lineage (Supplemental Fig. S3D). The transition from FL HSC to BM HSC is marked by further up-regulation of hematopoietic and immune genes, and down-regulation of cell cycle genes, metabolic genes, and several other classes of developmental genes (Supplemental Fig. S3F,G; Manesia et al. 2015).

### Diverse activity profiles of developmental enhancers during HSC ontogeny

Differential enhancer utilization is a key driver of cell fate change, and epigenetic patterning of enhancers often occurs before cell fate and subsequent gene expression changes. In order to identify potential regulatory elements and determinants of cell fate changes, we profiled cells along the HSC continuum by ChIP-seq for a combination of histone marks to identify regulatory elements, and discriminate between active (H3K4me1^+^, H3K4me3^−^, H3K27ac^+^, and H3K27me3^−^), and primed (H3K4me1^+^, H3K4me3^−^, H3K27ac^−^, and H3K27me3^−^) enhancers (Calo and Wysocka 2013). Using a low cell number protocol we previously developed for analyzing as few as 20,000 cells (Gao et al. 2019), we achieved an average sequencing depth of 29 million reads per sample, which is above the sequencing depth of 20 million reads recommended by the ENCODE consortium. Sixty-eight percent of total reads could be uniquely mapped to the mouse genome (Supplemental Fig. S4; Supplemental Table S4). The average (across four histone marks) genome-wide correlation of biological replicates was 0.957 (Supplemental Fig. S5).

**Figure 3.**
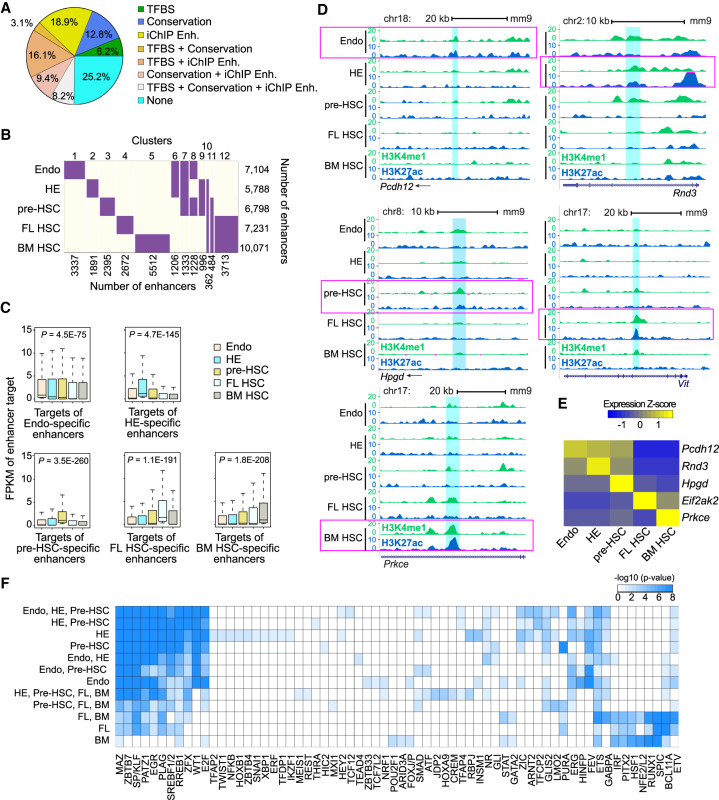
Repertoire of active enhancers during HSC ontogeny. (*A*) Supporting evidence for predicted enhancers. TFBS, transcription factor binding peaks from ChIP-seq data (Wilson et al. 2010); Conservation, conserved sequence across 20 mammalian genomes; iChIP Enh, enhancers from (Lara-Astiaso et al. 2014). (*B*) Clustering of enhancer activity profile across five developmental stages. (Purple) Active enhancers; (ivory) inactive enhancers. Numbers of enhancers in each cell type/cluster are shown. Only enhancers that are present in significant clusters are counted. (*C*) Box plots of expression profile of target genes of stage-specific enhancers. *P*-values, differential expression between stage-specific enhancer targets and targets of the rest of the enhancers using *t*-test. (*D*) Genome browser view of example developmental stage-specific enhancers (2-kb region highlighted in cyan) in Endo, HE, pre-HSC, FL HSC, and BM HSC. Magenta horizontal boxes highlight tracks for each cell population. (*E*) Target gene expression patterns of developmental stage-specific enhancers in *D*. (*F*) TFs whose DNA-binding motifs are enriched in stage-specific enhancers. Stage specificities are indicated at the *left* of each column. (BM) BM HSC; (FL) FL HSC. Color shade is proportional to the negative logarithm of enrichment *P*-value.

Using the chromatin signature identification by artificial neural network (CSI-ANN) algorithm (Firpi et al. 2010) and our epigenomic data, we predicted a total of 30,131 active enhancers (∼10,500 in each populations with some enhancers shared across stages; false discovery rate [FDR] < 0.05). Multiple lines of evidence corroborated our enhancer predictions. First, using additional purified cells, we confirmed the epigenetic signals of a select set of predicted enhancers by ChIP-qPCR (Supplemental Fig. S6). Second, our enhancer catalog is supported by additional public data, including sequence conservation across 20 mammalian genomes (34% of all predicted enhancers), a 53% overlap with reported enhancers in mouse BM HSCs and differentiated hematopoietic cell types (Lara-Astiaso et al. 2014), and 34% overlap with ChIP-seq peaks of ten hematopoietic TFs (Wilson et al. 2010). Overall, 75% of our predicted enhancers were supported by at least one line of evidence (Fig. 3A). The catalog also includes a number of well-known HSC enhancers validated by transgenic reporter assays in mice, such as the *Runx1* + 23 enhancer (Bee et al. 2009), the *Ly6a* (*Sca1*) enhancer (Ma et al. 2002), the *Tal1* (*Scl*) 3′ enhancer (Sánchez et al. 1999), the *Fli1* (Pimanda et al. 2007), *Gata2* (Lim et al. 2012), and *Erg* enhancers (Supplemental Table S5; Thoms et al. 2011).

Lara-Astiaso et al. (2014) used an epigenomics-based annotation strategy to catalog 48,391 enhancers in cell types spanning the developmental hierarchy from adult BM HSCs to terminally differentiated hematopoietic cell types such as lymphocytes and macrophages. To determine the difference between enhancers for HSC specification and differentiation, we intersected our enhancers (21,282, excluding Endo and BM HSC enhancers) with the enhancers by Lara-Astiaso et al. (2014) (37,548, excluding BM HSC enhancers). Strikingly, only 3020 enhancers are shared between the two data sets (Supplemental Table S6), suggesting that the enhancer repertoire for specifying HSC fate is quite distinct from that for multilineage differentiation from adult HSCs.

STEM clustering based on the enhancers’ activity profiles across the five developmental stages organized 83.4% (25,129) predicted enhancers into 12 clusters (*P* < 0.05, Fig. 3B). Enhancers that were active in all five stages, which only accounted for 2.1% of identified enhancers, were removed prior to clustering. Clusters 1–5 represent stage-specific enhancers active in Endo, HE, pre-HSCs, FL HSCs, or BM HSCs, accounting for 29.4% of all enhancers, demonstrating the dynamic transcriptional nature of HSC development. Cluster 6 represents enhancers uniquely active in Endo and HE. Enhancers in cluster 7 are active in Endo, HE and pre-HSCs. Enhancers in cluster 12 are only active in functional HSCs.

Using the integrated method for predicting enhancer targets (IM-PET) algorithm (He et al. 2014), we linked 25,541 active enhancers to their target promoters at the five developmental stages, for a total of 120,257 enhancer–promoter (EP) interactions (Supplemental Table S7). Expression levels of genes targeted by developmental stage-specific enhancers are significantly higher at the corresponding stage, supporting our EP predictions (Fig. 3C). On average, 2.3 promoters contact an enhancer in all populations (Supplemental Fig. S7A), while 4.4 enhancers contact a promoter (Supplemental Fig. S7B), consistent with results from recent large-scale chromatin interaction studies of EP interactions (Javierre et al. 2016; Chen et al. 2019). Only ∼14.3% of EP pairs had no intervening promoters from unrelated genes (Supplemental Fig. S7C). Thirty-five percent of predicted EP interactions were developmental stage-specific (Supplemental Fig. S7D). Figure 3D shows example stage-specific enhancers targeting protocadherin 12 (*Pcdh12*, Endo)*,* Rho family GTPase 3 (*Rnd3*, HE), prostaglandin dehydrogenase 1 (*Hpgd,* pre-HSC), translation initiation factor 2 α kinase (*Eif2ak2,* FL HSC, enhancer resides in intron of *Vit*), and protein kinase Cε (*Prkce,* BM HSC). The putative target genes of the enhancers exhibit the same developmental stage-specific expression patterns as their enhancers, consistent with regulation by the predicted enhancers (Fig. 3E).

To identify TFs that occupy the enhancers, we conducted a TF motif enrichment analysis using a set of 9500 enhancers from 18 nonhematopoietic cell types as the background (Supplemental Table S15). In total, we identified 78 enriched TF motif families across the 12 enhancer clusters (Fig. 3F). Motifs of many known hematopoietic TFs were identified, including RUNX1, GATA2, HEY2, LMO2, and HOXA9. Motifs of broadly expressed TFs such as Kruppel-like factors (KLFs) and specificity proteins (SPs) are also enriched at hematopoietic enhancers. The enriched motifs are organized into prominent groups. TF motifs on the left side of the plot, including, for example, MAZ, ZBTB7, SP/KLF, and PATZ2 are strongly enriched in the enhancers active in endothelial-like cells (Endo, HE, pre-HSC), become less enriched in FL HSC enhancers, and are not enriched in enhancers active in only BM HSCs (Fig. 3F). Most of the TFs that recognize these motifs are not thought of as canonical HSC TFs, but may be important during hematopoietic ontogeny in cells with endothelial-like properties. The opposite pattern is observed for TF motifs on the right side of the plot, which are more canonical HSC TF motifs that are not enriched in the enhancers active in Endo and HE cells. A small group of TF motifs are enriched only in active HE enhancers. Interestingly, two of the implicated HE-specific TFs, SNAI1 and TWIST1, are involved in endothelial to mesenchymal transitions (EndMT), a process similar to EHT.

### Repertoire of epigenetically primed enhancers during HSC ontogeny

**Figure 4.**
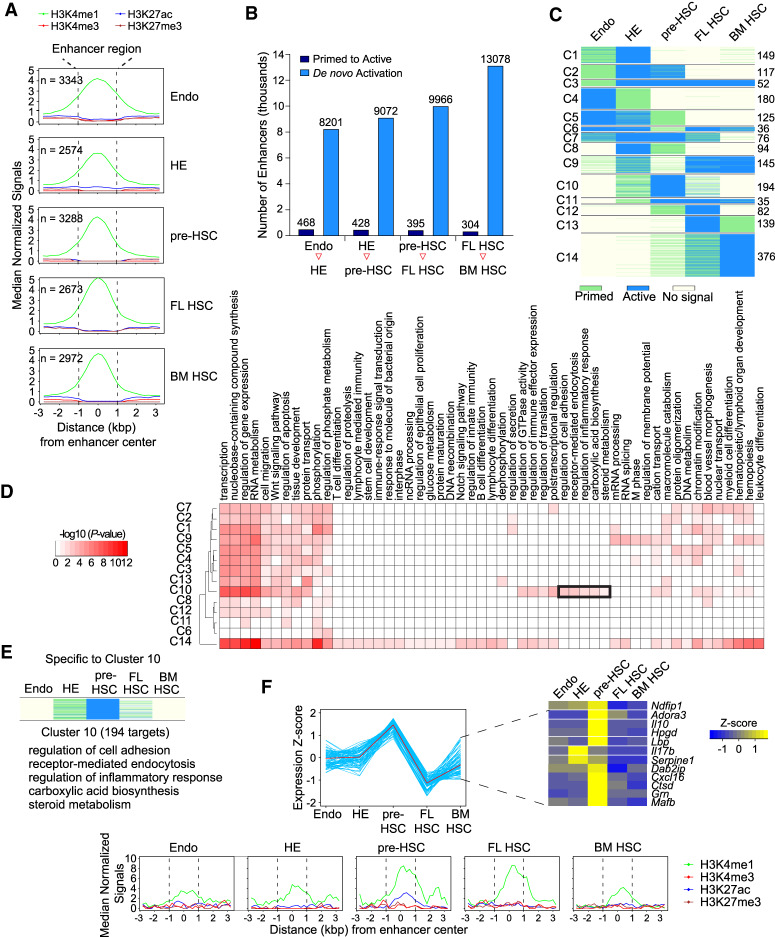
Primed enhancers during HSC ontogeny. (*A*) Metagene plot of normalized histone mark ChIP-seq signals at predicted primed enhancers (*n* indicates total number of primed enhancers identified in each stage) (*B*) Dynamics of primed and de novo activated enhancers during HSC ontogeny between each adjacent stage. (*C*) Clustering based on the activation pattern of the primed enhancers during HSC ontogeny. Cluster number is at the *left* and the number of enhancers within each cluster is at the *right*. (*D*) Enriched GO terms of genes targeted by primed enhancers within each cluster. A black rectangle highlights C10-specific GO terms specific for pre-HSCs. (*E*) Enhancer signal profile and enriched GO terms of predicted target genes of enhancers in cluster 10. (*F*, *top*) Expression of inflammatory response genes in cluster 10. (*Bottom*) Metagene plot of normalized histone mark ChIP-seq signals at primed enhancers targeting the inflammatory response genes in cluster 10.

Both active and epigenetically primed enhancers are marked by H3K4me1, but can be distinguished by the presence (active) or absence (primed) of H3K27ac, and absence of H3K27me3 and H3K4me3 modifications (Calo and Wysocka 2013). Primed enhancers play important roles in cell fate transitions during development and differentiation. Using the CSI-ANN algorithm and our epigenomic data, we predicted between 2574 and 3342 primed enhancers across the five developmental stages. The number of active enhancers is approximately 3.5 times greater than that of primed enhancers at all developmental stages (Fig. 4A; Supplemental Table S8). Enhancers can be activated with or without prior epigenetic priming; activation without priming is considered de novo activation. The two different activation mechanisms suggest different developmental functions of the enhancers. We found that the vast majority of hematopoietic enhancers are activated de novo without priming (Fig. 4B).

We next investigated the activation pattern of the primed enhancers during HSC ontogeny (Fig. 4C). We first identified primed enhancers that were activated either in the previous or subsequent developmental stage, and clustered these enhancers using their epigenetic profiles across the five stages. We observed diverse priming-and-activation patterns that can be grouped into 14 distinct clusters (Fig. 4C; Supplemental Table S9). Several groups consist of enhancers with a strong pattern of priming followed by activation (clusters C2, C3, and C12), or conversely, activated, then primed at a subsequent stage (clusters C4, C8, and C13). We also observed enhancers that gradually became primed or activated over several developmental stages (clusters C5 and C14). The different groups of enhancers regulate distinct gene pathways (Fig. 4D). Cluster C10 enhancers (*n* = 194) and their predicted target genes are of particular interest; they are primed in HE, become active in pre-HSCs, gradually lose their H3K27ac marks and revert to primed enhancers in FL HSCs, and subsequently lose their H3K4me1 marks and are deactivated in BM HSCs. The target genes within cluster C10, which are likely involved in regulating the expression of genes important for the specification of HE and formation of pre-HSCs, are enriched for several unique pathways, including regulation of cell adhesion, receptor-mediated endocytosis, regulation of inflammatory response, carboxylic acid biosynthesis and steroid metabolism (Fig. 4E). Several of these pathways, including inflammatory signaling, cholesterol biosynthesis, and vitamin D synthesis (i.e., steroid metabolism) were shown to promote the formation of HSPCs from HE (Li et al. 2014; Cortes et al. 2016; Gu et al. 2019). Consistent with the enhancer activation pattern, expression of the target pathways is also highest in pre-HSCs (Fig. 4F, left panel). For instance, expression of the inflammatory response genes is highest in pre-HSCs (Fig. 4F, right panel), these genes’ enhancers are primed in HE, and become active in pre-HSCs (Fig. 4F, bottom panel). In summary, our data suggest genes in multiple pathways are regulated by primed enhancers that respond to signaling pathways important for pre-HSC formation.

### Core transcriptional regulatory circuitries underlying developmental transitions

**Figure 5.**
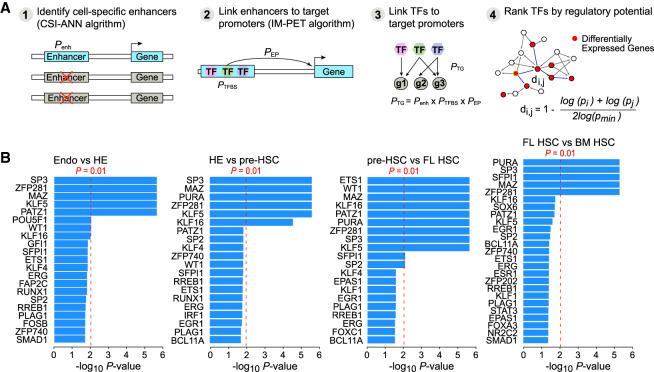
Developmental stage-specific transcriptional regulatory networks (TRNs) and key regulators in the networks. (*A*) Schematic for the computational framework for constructing the TRNs. The framework consists of four key steps: (1) identifying stage-specific enhancers using epigenetic signatures, (2) linking enhancers to target promoters using the IM-PET algorithm, (3) linking TFs to target promoters using a probabilistic scoring function *P*_*TG*_, and (4) identifying key TFs based on their regulatory potential on differentially expressed genes. (*B*) Key TFs predicted for four developmental transitions.

Little is known about the TRNs during HSC formation from arterial endothelium. We recently developed the target inference via physical connection (TIPC) algorithm for inferring condition-specific TRNs by integrating RNA-seq and histone mark ChIP-seq data (Gao et al. 2019). TIPC first computes probability scores for three key components of transcriptional regulation, including probability of a DNA sequence being an enhancer, probability of a TF binding to an enhancer based on the TF motif model and the enhancer sequence, and probability of enhancer–promoter interaction. The overall score for a TF regulating a target gene is the product of the three component probabilities (Fig. 5A). Using a set of gold standard TF target gene pairs in mouse embryonic stem cells, we demonstrated that TIPC achieves improved accuracy compared with several state-of-the-art methods (Gao et al. 2019). To increase robustness, we scanned the enhancer sequences for each TF motif using the Find Individual Motif Occurrence (FIMO) software (*P* < 1 × 10^−5^). We then applied TIPC to construct condition-specific TRNs for the five developmental stages. On average, each TRN consists of 381 TFs and 6530 target genes (Supplemental Table S10).

To identify key TFs regulating each developmental transition in HSC ontogeny, we merged developmentally adjacent TRN pairs and represented each gene in the merged network based on its level of differential expression during the transition. Next, we identified key TFs in the differential TRN using a network propagation-based method (Gao et al. 2019). The rationale for our method is that a key TF likely exerts its influence (either directly or indirectly) on the entire set of differentially expressed genes via a shorter regulatory path compared with paths from less important TFs. We predicted the sets of key TFs for each developmental transition using a heuristic method (Fig. 5B; Supplemental Material; Supplemental Fig. S8; Supplemental Table S10). Predicted TFs that were not expressed (FPKM = 0) at the particular developmental stage were removed from the final prediction. Expression levels of the predicted TFs across the developmental stages are shown in Supplemental Figure S9. TIPC predicted known regulators of HSC ontology, such as RUNX1 and GFI1, which are essential for EHT (Fig. 5B; North et al. 1999; Lancrin et al. 2012). Two other TFs predicted by TIPC, FOSB and SPI1, were sufficient to reprogram endothelial cells to HSCs when transiently expressed with RUNX1 and GFI1 (Sandler et al. 2014; Lis et al. 2017). Additional validated TFs that play a role in HE and/or HSPC formation predicted by TIPC were SP3, ETS1, ERG, and TFs belonging to the BMP-SMAD1 pathway (Van Loo et al. 2003; Wilson et al. 2010; Zhang et al. 2014). TIPC also predicted TFs that have yet to be implicated in HSC formation, such as Myc-associated zinc finger (MAZ). SP3 and MAZ were chosen to further validate because they were predicted to be regulators of multiple stages, and neither TF was known to regulate EHT, a step of interest for generating ex vivo HSCs.

### Novel roles of SP3 and MAZ in embryonic hematopoiesis

SP3 regulates fetal hematopoiesis in mice; germline deletion of *Sp3* resulted in hypocellularity of the fetal thymus and liver, a partial block at the CD4 CD8-double-positive stage of T-cell development, a decrease in the number of pre-B cells, and a delay in the appearance of definitive erythrocytes (Van Loo et al. 2003). The erythrocyte defect resulted from impaired erythrocyte differentiation and was not due to reduced EMP formation in the yolk sac. Fetal liver cells from E12.5 SP3-null embryos were able to engraft adult mice, but contributed to B and T cells only, and not to erythroid or myeloid lineage cells, hence, were defective in multilineage engraftment. All defects were identified in fetuses ranging in age from E12.5 to E18.5, from hematopoietic cells in organs colonized by HSPCs, and were not examined at the sites of HSPC origin. The impact of SP3 deletion was also analyzed in ES cell cultures, where it decreased the number of primitive erythroid progenitors (Gilmour et al. 2019). Whether SP3 played a role at the transitions predicted by TIPC, between Endo to HE, or HE to pre-HSCs, was not examined in either study (Van Loo et al. 2003; Gilmour et al. 2019). No role for MAZ in any aspect of hematopoiesis is reported.

**Figure 6.**
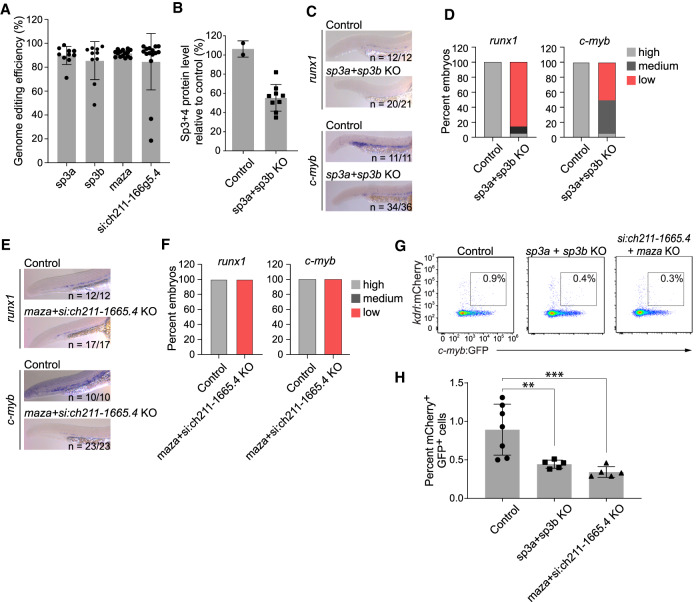
*Sp3* and *Maz* orthologs promote HSPC formation in zebrafish embryos. (*A*) Genome editing efficiency of each sgRNA determined using the tracking of indels by decomposition assay (TIDE). Data represent mean ± SD. *n* = 10 for *sp3a*; *n* = 10 for *sp3b*; *n* = 15 for *maza*; *n* = 15, for *si:ch211-166g5.4*. (*B*) Relative protein level of SP3 and SP4 in the knockout (KO) embryos. Because the only zebrafish antibody available is against both SP3 and SP4, the level of reduction of SP3 protein shown here is an underestimate. Data represent mean ± SD, *n* = 2 for-wild type embryos; *n* = 9 for *sp3* knockout embryos. (*C*) Representative whole-mount in situ hybridization (WISH) images for *runx1* and *c-myb* in the AGM region of 27 hpf and 30 hpf embryos, respectively. Embryos were injected with CRISPR guide RNAs targeting the *Sp3* orthologs *sp3a* and *sp3b*. The numbers of embryos with reduced *runx1* or *c-myb* expression relative to the number examined are indicated. (*D*) Bar graph depicting percentage of embryos with high, medium, and low *runx1* and *c-myb* expression following injection of sgRNAs targeting *Sp3* orthologs, and controls (summary of results in *C*). (*E*) Representative WISH images for *runx1* (27 hpf) and *c-myb* (30 hpf) in the AGM region of control and KO embryos injected with CRISPR guide RNAs targeting both *Maz* orthologs, *maza* and *si:ch211-166g5.4*. (*F*) Percentage of embryos with high, medium, and low reporter gene expression after injection of sgRNAs targeting the *Maz* orthologs. (*G*) Representative flow cytometry showing gating strategy for mCherry^+^ GFP^+^ HSPCs from embryos transgenic for *kdrl:mCherry* and *c-myb:GFP* reporter genes following KO of *Sp3* or *Maz* orthologs. (*H*) Frequency of mCherry^+^ GFP^+^ HSPCs in 30 hpf wild-type, *Sp3*, and *Maz* knockout embryos. *n* = 7 for wild-type embryos; *n* = 5 for *Sp3* embryos; *n* = 5, for *Maz* knockout embryos; number of experiments = 2. (***) *P* ≤ 0.01; (**) *P* ≤ 0.05.

To validate the predictive power of the TIPC algorithm to identify TFs important in hematopoietic ontogeny, we used CRISPR-Cas9 to knock out the orthologs of *Sp3* and *Maz* in zebrafish embryos. We focused on the impact of the mutations on HE, since this is the first step in hematopoietic ontogeny for which a role for SP3 and MAZ was predicted. The cellular and genetic programs mediating HE specification are highly conserved in mice and zebrafish (Carroll and North 2014). *Runx1*^+^ HE cells in zebrafish embryos appear in the dorsal aorta at 22 h postfertilization (hpf), and the first *c-myb*^+^ cells (equivalent to IAC cells) differentiate from HE cells at 25 hpf. HSPCs expressing both *kdrl* and *c-myb* then migrate to and seed the caudal hematopoietic tissue (CHT), the mouse fetal liver equivalent, at 48 hpf. Both *Sp3* and *Maz* have two orthologs in zebrafish (*sp3a* and *sp3b* for *Sp3*, *maza* and *si:ch211-166g5.4* for *Maz*), and we knocked out all four genes (Fig. 6A,B; Supplemental Tables S11, S12). The editing frequency for each gene was at least 85.5%, although total protein levels for the *Sp3* knockout were reduced by only 50% as the antibody used in the Western blot also recognizes SP4 (Fig. 6A,B). *Runx1* and *c-Myb* expression in the dorsal aorta peaked in control embryos at 27 hpf and 30 hpf, respectively (Supplemental Fig. S10); knockout of *Sp3* (*sp3a* + *sp3b*) decreased *Runx1* and *c-Myb* expression (Fig. 6C,D), as did knockout of the two *Maz* orthologs (Fig. 6E,F). The percentage of mCherry^+^ GFP^+^ HSPCs in 30-hpf *Tg(kdrl:mCherry) Tg(myb:GFP)* embryos was also significantly reduced in both *Sp3* and *Maz* knockout embryos (Fig. 6G,H). To exclude the possibility that reductions in HE and HSPCs were due to antecedent vascular abnormalities, we examined the integrity of blood vessels in *Tg(kdrl:mCherry)* transgenic embryos at 48 hpf when intersegmental blood vessels (ISVs) are present (Tamplin et al. 2015). There were no significant differences in the vascular sprouting patterns, the function of ISVs, or in the diameters of the ISVs, dorsal aortae, or posterior cardinal veins between wild-type and knockout embryos of either genotypes, indicating the decreases in *Runx1* and *c-Myb* expression in *Sp3* and *Maz* knockout embryos resulted from specific defects in HE and HSPCs (Supplemental Fig. S11).

We next analyzed mouse embryos to confirm SP3's role in specifying HE. SP3-null embryos on a C57BL/6 background die between E14.5 and E18.5 due to cardiac malformations (Van Loo et al. 2007). However, on a mixed C57BL/6 and 129/Ola background, SP3-null mice survived until birth and died from respiratory failure (Bouwman et al. 2000). Since the emergence of HSPCs is dependent on blood flow (North et al. 2009), we generated SP3-null mice on a mixed background (C57BL/6x B6129SF1/J) to ameliorate potential vascular defects resulting from compromised cardiac function. At E10.5, SP3-null embryos displayed no gross vascular abnormalities (data not shown). Close examination of the yolk sac vasculature, the remodeling of which is known to be sensitive to alterations in hemodynamic forces (Lucitti et al. 2007) revealed a hierarchical vasculature structure with vessels of normal caliber, indicating that hemodynamic forces were not appreciably altered (Supplemental Fig. S12A,B).

**Figure 7.**
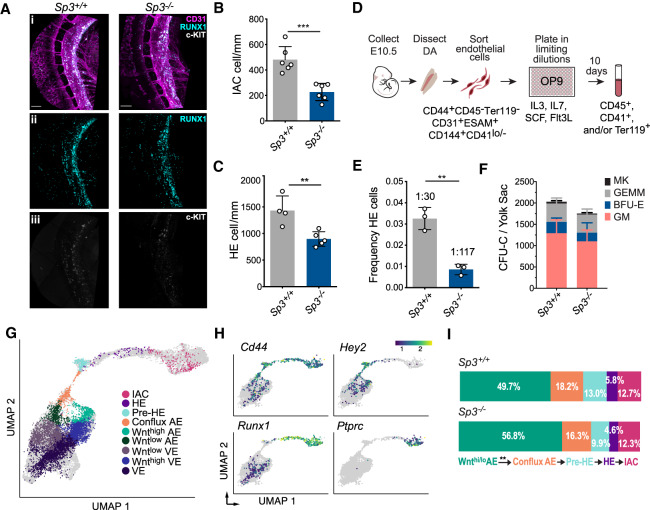
*SP3* regulates the frequency of HE cells in the dorsal aorta of mouse embryos. (*A*) Confocal z-projections (z-intervals = 2.5 µm) of dorsal aortas from E10.5 *Sp3*^+/+^ and *Sp3*^−/−^ mouse embryos. Embryos were immunostained for CD31 (panel *i*), RUNX1 (panels *i*,*ii*) and c-KIT (panels *i*,*iii*). Scale bars, 100 µm. (*B*) Quantification of CD31^+^RUNX1^+^c-KIT^+^ intra-aortic hematopoietic cluster cells in the lumen of the dorsal aortas of E10.5 embryos (mean ± SD, unpaired two-tailed *t*-test). (*C*) Quantification of HE cells (CD31^+^RUNX1^+^c-KIT^−^) in the wall of the dorsal aorta (mean ± SD, unpaired two-tailed *t*-test). (*D*) Illustration of a limiting dilution assay to determine the frequency of functional HE cells in a purified population of CD44^+^ endothelial cells enriched for HE. (*E*) Frequency of HE cells in CD44^+^CD45^−^Ter119^−^CD31^+^ESAM^+^CD144^+^CD41^lo/−^ cells purified from the dorsal aortas of E10.5 *Sp3*^+/+^ or *Sp3*^−/−^ embryos (mean ± SD, unpaired two-tailed *t*-test). Each replicate consisted of pooled cells from separate litters of E10.5 embryos collected in independent experiments. (***) *P* ≤ 0.001; (**) *P* ≤ 0.01. (*F*) Colony-forming unit cells (CFU-Cs) per E10.5 YS (mean ± SD). (MK) Megakaryocyte; (GEMM) granulocyte macrophage–erythroid–megakaryocyte; (BFU-E) erythroid blast-forming unit; (GM) granulocyte macrophage. Data are from three independent litters. (*G*) Projection of combined *Sp3^+/+^* and *Sp3^−/−^* cells onto the EHT trajectory from Zhu et al. (2020). Cell types were annotated using a k-nearest-neighbor classifier (see Supplemental Material). AE is defined by gene module scoring of arterial-specific genes (*Efnb2*, *Nrp1*, *Gja5*, *Bmx*, *Notch1*, *Notch4*, *Dll4*, *Jag1*, *Jag2*, *Acvrl1*, *Epas1*, *8430408G22Rik*, and *Vegfc*) relative to venous-specific genes (*Ephb4*, *Flt4*, *Nrp2*, *Nr2f2*, and *Emcn*) as described in Zhu et al. (2020). (*H*) Expression pattern of selected cell type-specific genes on the UMAP plot. (*I*) Fraction of Wnt^high/low^ AE, conflux AE, pre-HE, HE, and IAC cells in Ter119^−^CD41^lo/−^CD31^+^CD144^+^ESAM^+^ cells purified from E10.5 *Sp3^+/+^* versus *Sp3^−/−^* embryos. Only the difference in the proportion of cells in Wnt^high/low^ AE versus conflux AE was significant (*P* < 0.006, proportion test).

We enumerated IACs and HE cells in E10.5 SP3-null embryos, when the number of IACs peaks, by whole-mount confocal microscopy. Loss of SP3 reduced the number of CD31^+^RUNX1^+^c-KIT^+^ IACs in the lumen, and CD31^+^RUNX1^+^c-KIT^lo/−^ HE cells in the wall of the dorsal aorta, consistent with a role for SP3 in specifying HE (Fig. 7A–C). SP3 loss also decreased the frequency of functional HE cells. We purified a population of CD44^lo/+^ endothelial cells enriched for HE (Oatley et al. 2020) from the dorsal aorta of E10.5 embryos and determined the frequency of HE cells within that population ex vivo (Fig. 7D). The frequency of HE cells in SP3-null embryos was reduced by 75% compared with wild-type embryos (Fig. 7D,E), indicating that SP3 promotes the specification of HE cells in the dorsal aorta of mouse embryos.

To determine when during the specification of HE SP3 is required, we purified cells with endothelial markers from the dorsal aorta of E10.5 embryos and performed single cell RNA-seq (scRNA-seq). We recently showed that cells expressing endothelial markers form a continuous trajectory in a uniform manifold approximation projection (UMAP) plot, with several identifiable transitional stages (Fig. 7G; Zhu et al. 2020). HE is derived from arterial endothelium (AE), which we defined by higher expression of arterial-specific genes (e.g., *Efnb2, Nrp1, Dll4*) relative to venous-specific genes (e.g., *Ephb4*, *Nrp2*, and *Nr2f2*) (Zhu et al. 2020). The AE cells form two streams in the UMAP trajectory, one containing cells expressing high levels of Wnt target genes (Wnt^hi^ AE), and the other expressing lower levels of Wnt target genes (Wnt^lo^ AE). Wnt^hi^ and Wnt^lo^ AE converge to form a population of CD44^+^ AE cells called “conflux AE,” which in turn gives rise to prehemogenic endothelial cells (pre-HE) expressing high levels of Notch target genes (e.g., *Hey2*) (Fig. 7G,H). A portion of pre-HE cells differentiate into *Runx1*^+^HE cells, which then form *Ptprc*^+^IACs (Fig. 7G,H). We examined how SP3 loss affected the distribution of cells between each of the stages from Wnt^hi/lo^AE cells to IACs. scRNA-seq data of cells expressing endothelial markers purified from the dorsal aorta of E10.5 SP3 wild-type and null embryos, mapped onto the UMAP plot of our previous data, revealed a significant increase in the proportion of Wnt^hi/lo^ AE cells in SP3-null embryos, and a corresponding decrease in conflux AE and subsequent populations, suggesting that SP3 regulates the transition from Wnt^hi/lo^AE to conflux AE (Fig. 7I). We did not observe significant inefficiencies at later steps in the trajectory, but cannot rule them out, since the small numbers of cells at later stages were likely insufficient to detect small perturbations in their distribution (Supplemental Table S16). In summary, the scRNA-seq data indicate that SP3 regulates the efficiency of HE specification starting at a relatively early stage, at the transition between Wnt^hi/lo^ AE cells and conflux AE, in contrast to the perturbation caused by haploinsufficiency of Runx1 that occurs at the pre-HE to HE transition (Zhu et al. 2020).

To confirm that the inefficiency in HE specification in SP3-null embryos was not due to defects in blood flow, we analyzed the scRNA-seq data for the expression of several flow-regulated genes. There was no significant difference in the expression of *Nos3* (eNOS) (Supplemental Fig. S12C), encoding nitric oxide synthase 3, which is regulated by laminar shear stress (Topper et al. 1996). The expression of other genes regulated by flow including *Cav1, Nrf2*, and *Klf2* was also unaffected by SP3 loss. Therefore, as in the zebrafish embryo, any defects in HSPC formation in SP3-null mouse embryos were not caused by secondary effects from cardiac abnormalities and impaired blood flow. Interestingly, loss of SP3 had no effect on EMP formation in the yolk sac (Fig. 7F), confirming a previous study (Van Loo et al. 2003), indicating that SP3 does not regulate the specification of yolk sac HE, but is required only in arterial HE. This further validates the ability of TIPC to predict TFs required for definitive hematopoiesis in the major arteries of the embryo.

## Discussion

Here we provide the first comprehensive map of the transcriptome and the epigenome throughout HSC ontology from the major arteries. We defined transcriptional enhancers that are primed or activated at five distinct stages of endogenous definitive hematopoiesis in the AGM. Thirty percent of the identified enhancers are specifically active at one developmental stage, which exceeds the percentage of differentially expressed genes (12%) between any two adjacent developmental stages. These results suggest that the *cis*-regulatory landscape is more dynamic than gene expression during HSC ontogeny, and that enhancer patterns may be finer-grained descriptors of cellular differentiation stages than gene expression. It is possible that the excess of stage-specific enhancers relative to stage-specific gene expression may be driven by a need for combinatorial regulation by multiple enhancers to achieve specific complex expression patterns. Alternatively, or in addition, an inherent redundancy may be built into the regulatory program to achieve evolutionary robustness.

Enhancer priming enables fast and sustained transcriptional responses to signaling, but it is not a prerequisite for the establishment of active enhancers. Lara-Astiaso et al. (2014) found that 40% of enhancers are established de novo during the differentiation from HSCs to mature hematopoietic cells, and the establishment of de novo lineage-specific enhancers occurs mainly at the root of the commitment point in the first progenitor of the lineage. Studies on B-cell development support this finding and also show that early priming makes only a minor contribution to enhancer repertoire establishment during B-cell development (Luyten et al. 2014). In our study, de novo activation is the primary method (>94.6% for all active enhancers), suggesting that it plays a major role in hematopoietic ontogeny.

Our study confirmed the importance of the broadly expressed TF SP3 in HSC ontogeny (Van Loo et al. 2003), and identified a role for the related protein MAZ. Validation of other predicted TFs or combination of TFs will be the focus of future studies. The TIPC algorithm predicted a role for SP3 at several stages of hematopoietic ontogeny including in HE, and indeed SP3 loss decreased the number of phenotypic and functional HE cells. However, the scRNA-seq data indicate that the reduction in HE cells is largely due to inefficiency at a transition within the heterogeneous Endo population, between Wnt^hi/lo^ AE and conflux AE. Interestingly, scRNA-seq detected very few differentially expressed genes in WT and SP3 KO endothelial cells (data not shown), suggesting that SP3 may mediate very small alterations in the expression of a large number of genes that do not cross the threshold of detectability. TIPC also predicted a role for SP3 in the transition from HE to pre-HSCs but scRNA-seq did not confirm this prediction, either because the number of HE and IAC cells captured by scRNA-seq was too small to detect a difference (Supplemental Table S16), or that the E11.5 pre-HSCs used in the TIPC analysis are not equivalent to the E10.5 IAC cells profiled by scRNA-seq.

SP3 and MAZ are zinc finger proteins that recognize similar, although distinct, GC-rich DNA sequences (Van Loo et al. 2007). Twenty seven percent of human promoters contain GC-rich regions, making GC boxes the most prevalent promoter motifs (FitzGerald et al. 2004). The ubiquitously expressed TFs that bind GC boxes are capable of regulating a large number of genes involved in fundamental cellular processes including proliferation, apoptosis, migration, adhesion, and angiogenesis. The tissue-specific activity of these TFs is determined by their interaction with lineage-specific TFs. For example, SP3 was reported to bind regions occupied by RUNX1 and PU.1 in myeloid cells (Lennartsson et al. 2003), and SP1 has been reported to physically interact with RUNX1 (Wei et al. 2008), GATA2 (Maeda et al. 2010), and the SCL complex consisting of SCL, LMO2, and GATA1/2 (Lécuyer et al. 2002).

The cell type-specific activity of broadly expressed TFs could be conferred by the chromatin landscape established by lineage-specific TFs. This model is supported by data showing that loss of SP1 in ES cells had little effect on chromatin structure, and that SP1 and SP3 primarily bind in regions of accessible chromatin marked by the presence of H3K27ac and H3K4me3, and the absence of H3K9me3 (Gilmour et al. 2019). Our data fit with the model proposed by Gilmour et al. (2019) suggesting that ubiquitous factors, such as SP3 and MAZ, are important for establishing a stable promoter structure that interacts with distal regulatory elements. In this model, lineage-specific factors are responsible for making the correct distal regulatory elements accessible for ubiquitously expressed factors to interact with.

The stage-specific enhancers and core transcriptional regulatory circuitries identified in this study will be an important resource for isolating transitional cell populations during hematopoietic ontogeny, as well as for the de novo generation of HSCs from induced pluripotent stem cells (iPSCs) or other cell sources. Currently, the majority of protocols for deriving HSCs from iPSCs recapitulate yolk sac hematopoiesis, which produces a different complement of HSPCs than the major arteries (Slukvin and Uenishi 2019). Protocols for ex vivo HSPC production could be improved by a more comprehensive understanding of endogenous definitive hematopoiesis in the major arteries, and of the differences between HE in both sites. In addition, previous attempts to reprogram endothelial cells to become HSCs used TFs specifically expressed in HSCs versus endothelial cells (Lis et al. 2017). The efficiency of this process could potentially be improved by also introducing TFs normally expressed during HSC ontogeny in the arterial endothelial cell precursors of HSCs.

## Materials and methods

### Mouse strains

Hemogenic and nonhemogenic endothelial cells were purified from *Runx1*^*tm4Dow*^ embryos (Lorsbach et al. 2004). Pre-HSCs and embryonic HSCs were isolated from B6.Cg-Tg(Ly6a-GFP)G5Dzk/J embryos. Female B6C3F1/J mice were mated with male B6129SF1/J mice to generate E14.5 fetuses for purifying fetal liver HSCs. Pairs of 6- to 8-wk-old female and male B6C3F1/J and B6129SF1/J mice were used to isolate bone marrow HSCs. *Sp3* mutant mice (B6.129P2-Sp3^tm1Sus^/Cnrm) were obtained from the EMMA repository (EM:02429) and genotyped as described in Krüger et al. (2007). We crossed C57BL6 *Sp3*^+/−^ males to B6129SF1/J wild-type females (The Jackson Laboratory 101043) to obtain F1 *Sp3*^+/−^ females, with which we performed timed matings with C57BL6 *Sp3*^+/−^ males to obtain *Sp3*^−/−^ embryos for analysis. The University of Iowa and University of Pennsylvania Offices of the Institutional Animal Care and Use Committee (IACUC) review boards approved these studies. This study was performed in accordance with the recommendations in the Guide for the Care and Use of Laboratory Animals of the National Institutes of Health. All of the animals were handled according to approved IACUC protocols.

### Low cell number ChIP-seq

ChIP-seq was done using a low cell number ChIP-seq protocol developed by us (Gao et al. 2019). About 2000 cells were used per IP and libraries were prepared using the ThruPLEX-FD preparation kit or ThruPLEX DNA-seq (Rubicon Genomics). Libraries were sequenced on an Illumina HiSeq 2500 sequencer in single-end mode with read length of 50 nt.

### RNA sequencing

Cells were sorted into TRIzol-LS (Invitrogen) and total RNA was purified using RNeasy micro kit (Qiagen) and analyzed for integrity using RNA 6000 pico kit for 2100 Bioanalyzer (Agilent). mRNA was purified from total RNA using NEBNext Poly(A) mRNA magnetic isolation module (NEB). Poly(A)-selected mRNA was fragmented to an average size of 300 nt, reverse-transcribed, and converted to a paired-end sequencing library using NEBNext Ultra RNA library preparation kit for Illumina (NEB) according to the vendor's instructions. Libraries were sequenced on an Illumina HiSeq 2500 sequencer in paired-end mode with the read length of 75 nt.

### Reagents

All antibodies used in this study are listed in Supplemental Table S13. All PCR primers are listed in Supplemental Table S14.

### Data availability

All data generated in this study has been deposited in the Gene Expression Omnibus (GEO) database under the accession number GSE135601.

## Supplementary Material

Supplemental Material
