# Supplementary material for: Transcriptional regulatory network controlling the ontogeny of hematopoietic stem cells
Source: Genes Dev. 2020 Jul 1;34(13-14):950–64. doi: 10.1101/gad.338202.120 (PMC7328518; doi:10.1101/gad.338202.120)
Supplement: Supplemental Material [file supp_34_13-14_950__index.html]

Transcriptional regulatory network controlling the ontogeny of hematopoietic stem cells — Supplemental Material 

# Transcriptional regulatory network controlling the ontogeny of hematopoietic stem cells

## Supplemental Material

- Supplemental\_Figs.zip
- Supplemental\_Fig\_S4.pdf
- Supplemental\_Fig\_S5.pdf
- Supplemental\_Fig\_S6.pdf
- Supplemental\_Fig\_S7.pdf
- Supplemental\_Fig\_S8.pdf
- Supplemental\_Fig\_S10.pdf
- Supplemental\_Fig\_S11.pdf
- Supplemetnal\_Materials-EH.docx
- Supplemental\_Table\_S1-NEW.docx
- Supplemental\_Table\_S2-NEW.docx
- Supplemental\_Table\_S3\_NEW.xlsx
- Supplemental\_Table\_S4.docx
- Supplemental\_Table\_S5.docx
- Supplemental\_Table\_S6.xlsx
- Supplemental\_Table\_S7.xlsx
- Supplemental\_Table\_S8.xlsx
- Supplemental\_Table\_S9.xlsx
- Supplemental\_Table\_S10.xlsx
- Supplemental\_Table\_S11.docx
- Supplemental\_Table\_S12.docx
- Supplemental\_Table\_S13.docx
- Supplemental\_Table\_S14.docx
- Supplemental\_Table\_S15.xlsx
- Supplemental\_Table\_S16.docx
