# Supplementary figures and images for: Transcriptional regulatory network controlling the ontogeny of hematopoietic stem cells
Source: Genes Dev. 2020 Jul 1;34(13-14):950–64. doi: 10.1101/gad.338202.120 (PMC7328518; doi:10.1101/gad.338202.120)

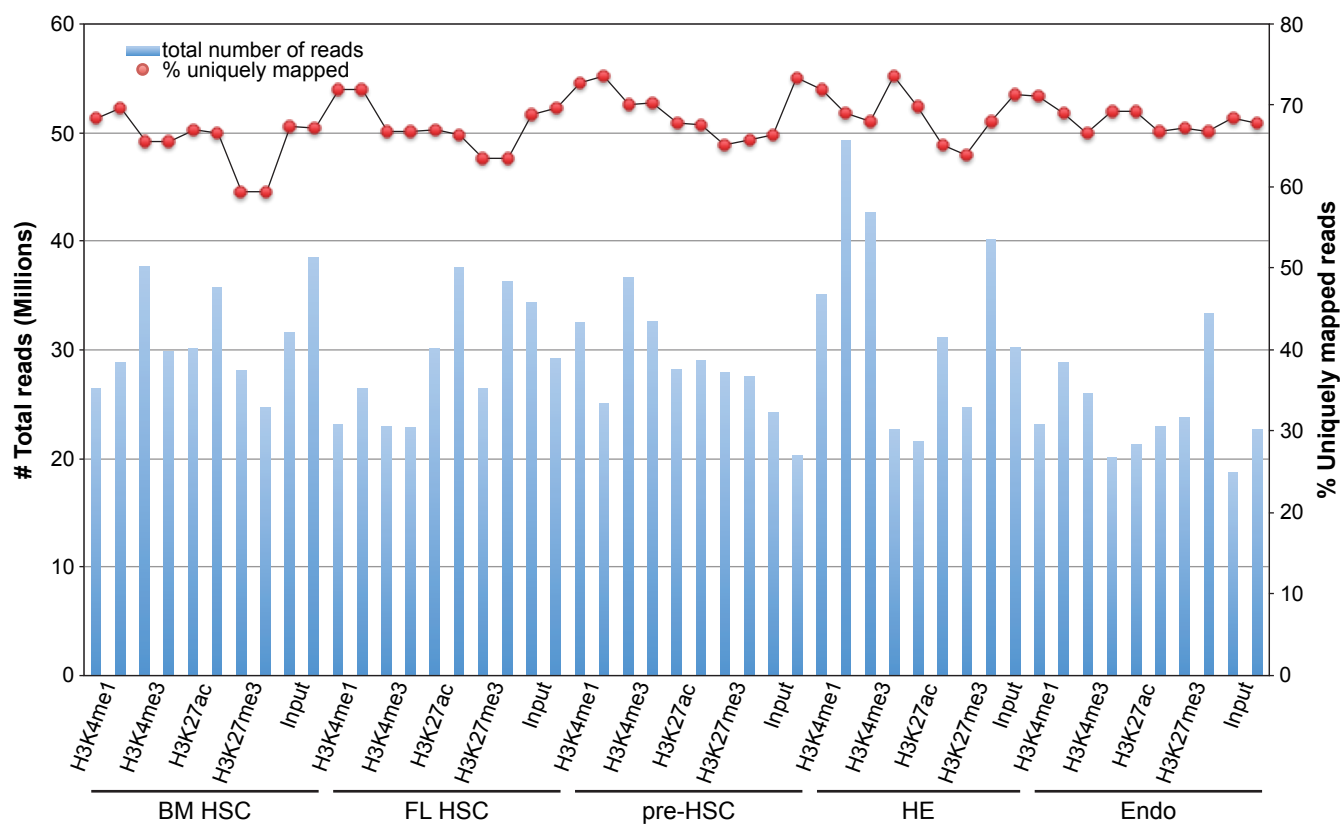

Supplement: Supplemental Material [file supp_gad.338202.120_Supplemental_Fig_S4.pdf]

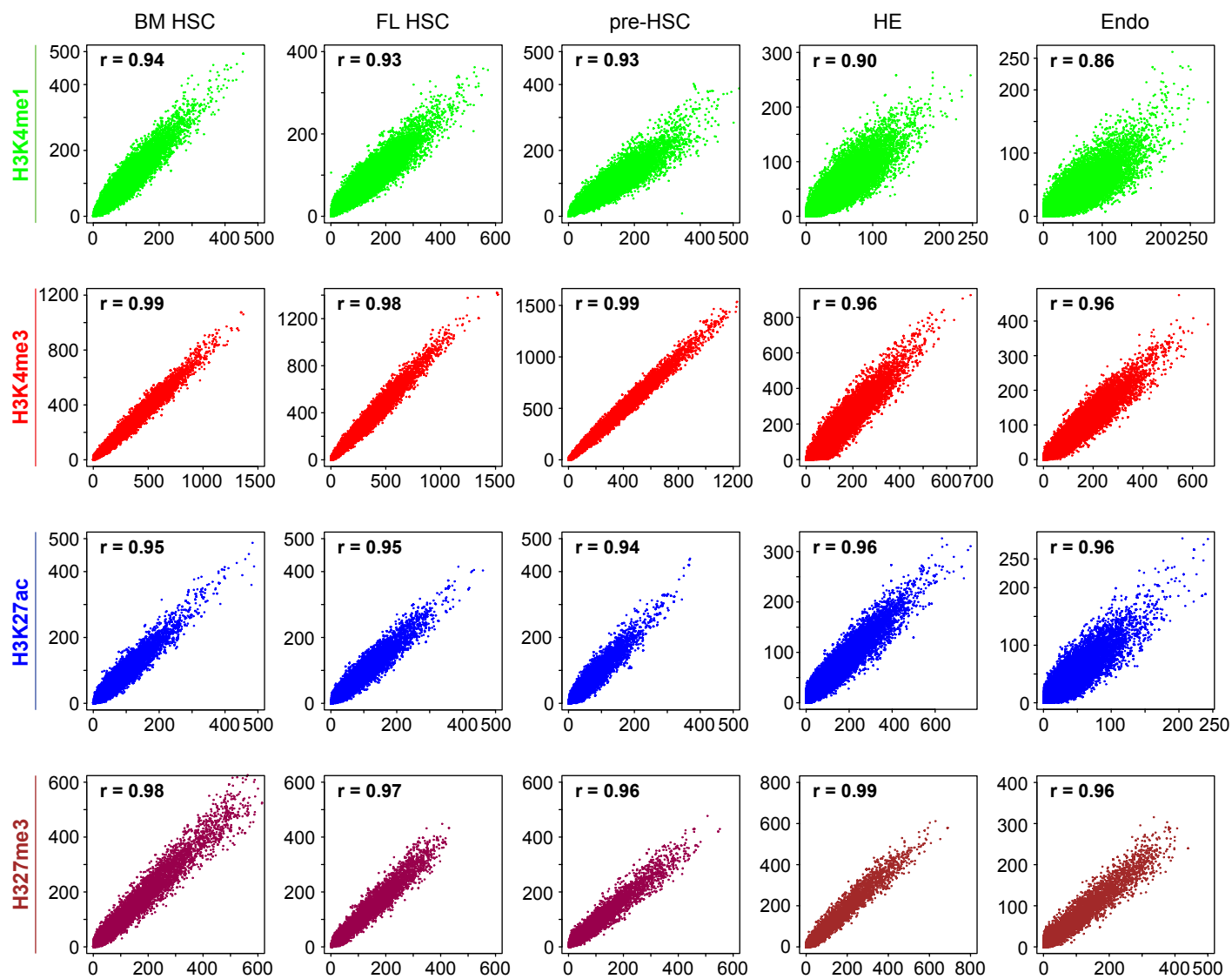

Supplement: Supplemental Material [file supp_gad.338202.120_Supplemental_Fig_S5.pdf]

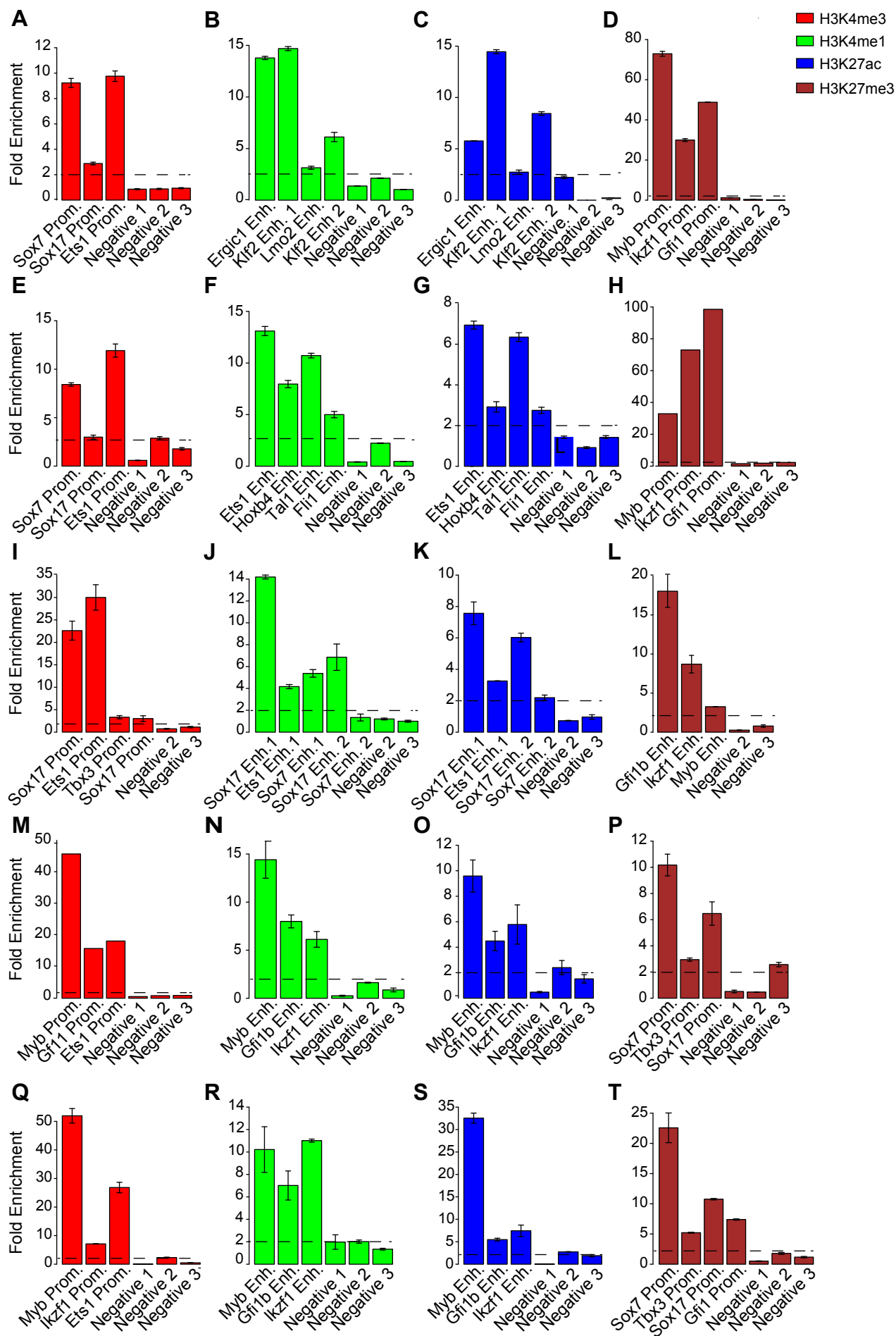

Supplement: Supplemental Material [file supp_gad.338202.120_Supplemental_Fig_S6.pdf]

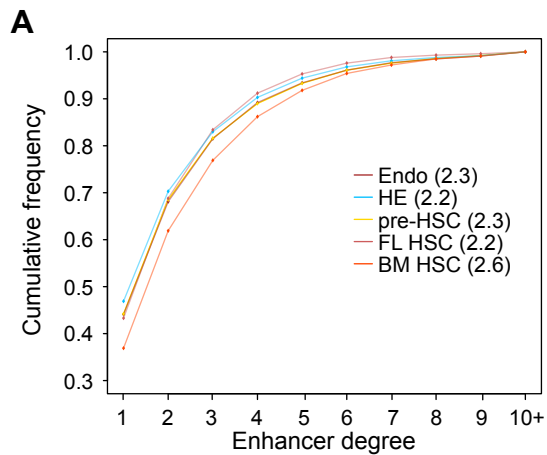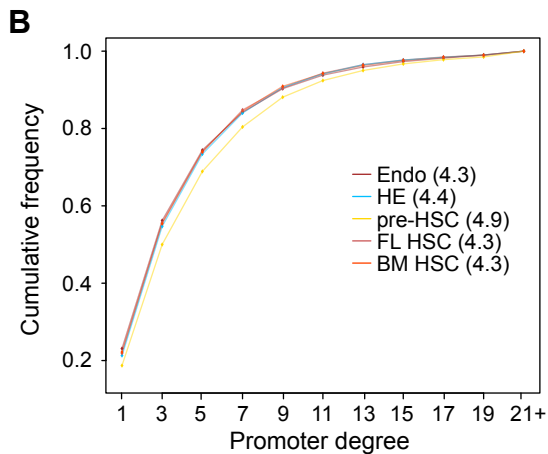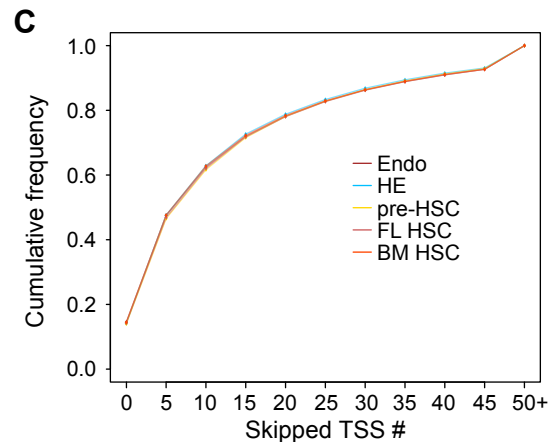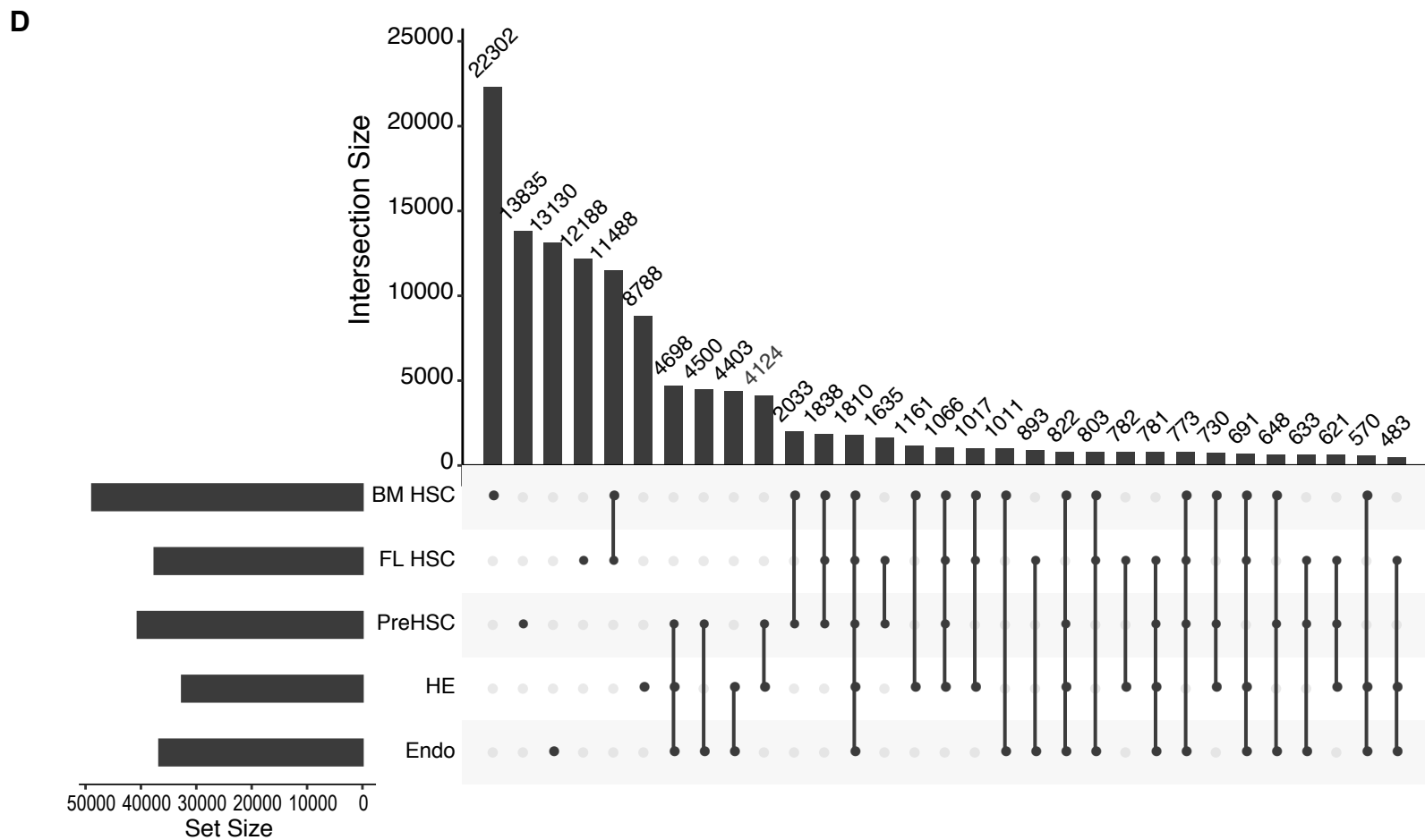

Supplement: Supplemental Material [file supp_gad.338202.120_Supplemental_Fig_S7.pdf]

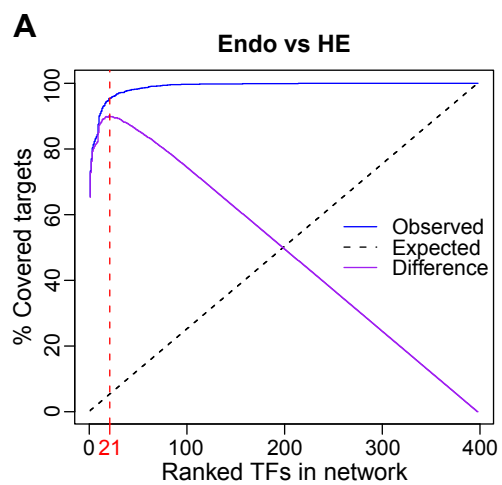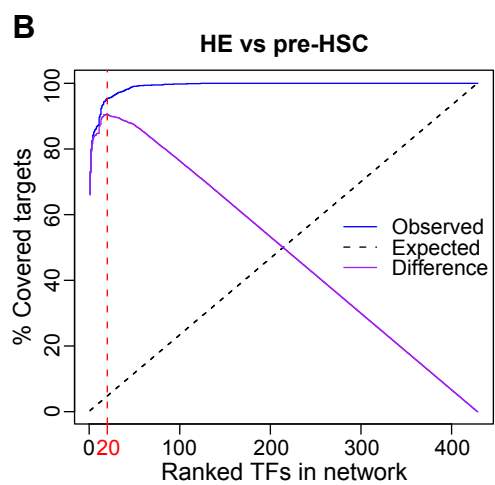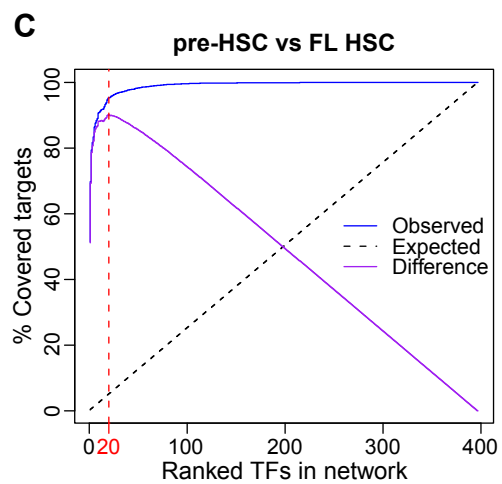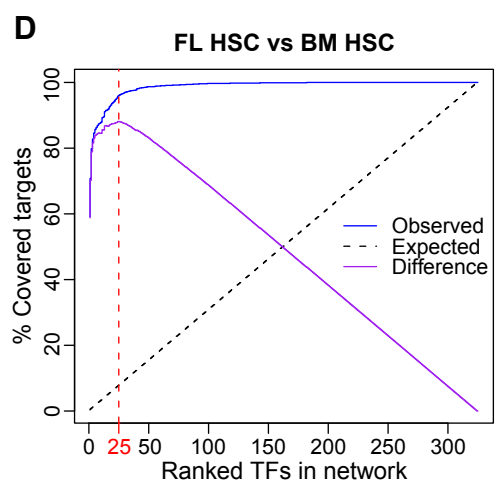

Supplement: Supplemental Material [file supp_gad.338202.120_Supplemental_Fig_S8.pdf]

*Runx1*

*c-myb*

24 hpf

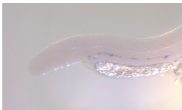

27 hpf

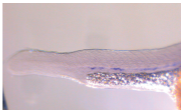

27 hpf

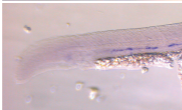

30 hpf

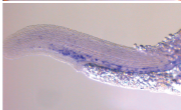

30 hpf

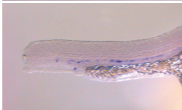

33 hpf

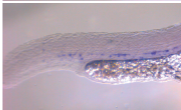

33 hpf

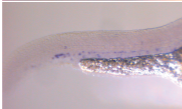

36 hpf

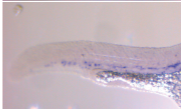

Supplement: Supplemental Material [file supp_gad.338202.120_Supplemental_Fig_S10.pdf]

**A**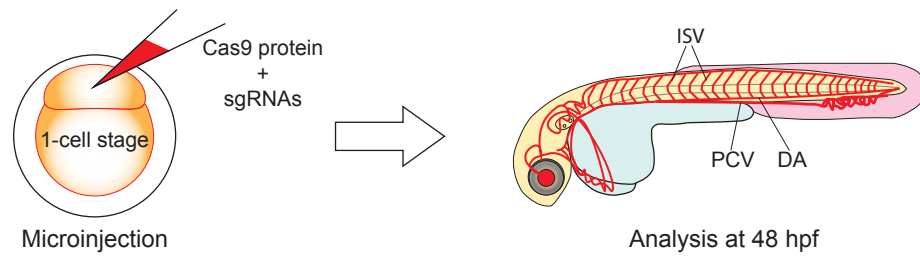**B**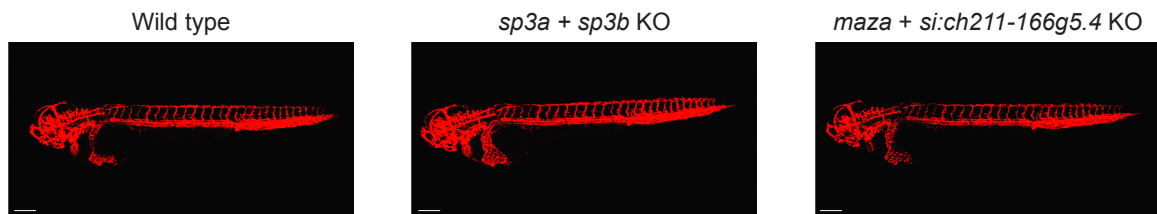**C**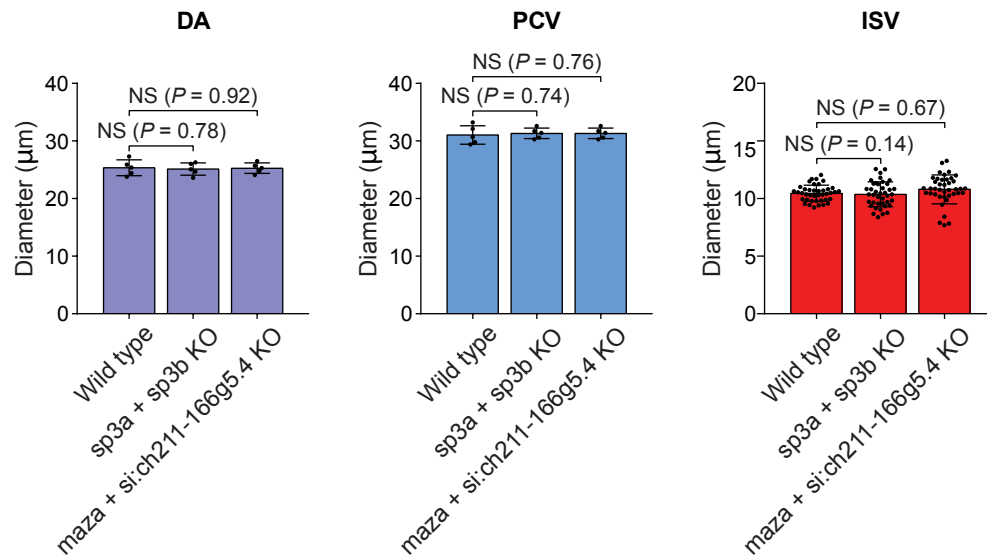

Supplement: Supplemental Material [file supp_gad.338202.120_Supplemental_Fig_S11.pdf]
