# Supplementary material for: Transcriptional regulatory network controlling the ontogeny of hematopoietic stem cells
Source: Genes Dev. 2020 Jul 1;34(13-14):950–64. doi: 10.1101/gad.338202.120 (PMC7328518; doi:10.1101/gad.338202.120)
Supplement: Supplemental Material [file supp_gad.338202.120_Supplemental_Material_.docx]

**Transcriptional regulatory network controlling the ontogeny of hematopoietic stem cells**

Peng Gao^1,10^, Changya Chen^1,10^, Elizabeth D. Howell^2,3,10^, Yan Li^2,8,10^, Joanna Tober^2^, Yasin Uzun^1^, Bing He^1,9^, Long Gao^4^, Qin Zhu^5^, Arndt Siekmann^2^, Nancy A. Speck^2,6,*^, and Kai Tan^1,2,4,7,*^

^1^ Division of Oncology and Center for Childhood Cancer Research, Children’s Hospital of Philadelphia, Philadelphia, PA 19104, USA

^2^ Department of Cell and Developmental Biology, Perelman School of Medicine, University of Pennsylvania, Philadelphia, PA 19104, USA

^3^ Graduate Group in Cell and Molecular Biology, University of Pennsylvania, Philadelphia, PA 19104, USA

^4^ Department of Genetics, Perelman School of Medicine, University of Pennsylvania, Philadelphia, PA 19104, USA

^5^ Graduate Group in Genomics and Computational Biology, University of Pennsylvania, Philadelphia, PA 19104, USA

^6^ Abramson Family Cancer Research Institute, Perelman School of Medicine, University of Pennsylvania, Philadelphia, PA 19104, USA

^7^ Department of Pediatrics, Perelman School of Medicine, University of Pennsylvania, Philadelphia, PA 19104, USA

^8^ Present address: Department of Veterinary Medicine and Institute of Preventive Veterinary Sciences, Zhejiang University College of Animal Sciences, Hangzhou, Zhejiang 310058, China

^9^ Present address: Department of Pediatrics, College of Medicine, Pennsylvania State University, Hershey, PA 17033

^10^These authors contributed equally to this work.

^*^Corresponding author

**Supplemental Materials**

**Supplemental Methods**

**Tissue dissection and cell purification using fluorescence-activated cell sorting**

Hemogenic and non-hemogenic endothelial cells: Embryos were collected from B6C3F1 female mice mated to *Runx1^tm4Dow^* male mice and dissected in 1× PBS + 10% FBS + 1% Penicillin-Streptomycin (Gibco). AGM regions, umbilical and vitelline vessels were separately collected and digested in 0.125% collagenase (Sigma) for 1 hour at 37 °C. Cells were washed and stained in an antibody cocktail against CD41, CD31, c-Kit, CD45 (eBiosciences) and 7-AAD or DAPI (Invitrogen). Cells were sorted on a Becton Dickinson (BD) FACS Jazz (BD Biosciences) or BD Influx into 1× PBS + 20% FBS + 25 mM HEPES in a 1.5 mL LoBind tube.

Pre-HSCs: 5 IU of pregnant mare serum gonadotropin (Sigma) and 5 IU of human chorionic gonadotropin (Sigma) were injected into 3 weeks old B6C3F1/J female mice by intraperitoneal injection to stimulate superovulation, and mated to (Ly6a:GFP) male mice [B6.Cg-Tg(Ly6a-GFP)G5Dzk/J] [(Ma et al. 2002)](https://paperpile.com/c/POiN9L/D4NL). AGM, vitelline and umbilical arteries were dissected from E11.5 Ly6a:GFP^+^ embryos. Pooled tissues were dissociated with collagenase (Sigma). Cells were washed and resuspended in 1× PBS + 10% FBS (Gibco) and stained with antibodies against CD31, c-Kit, ESAM and CD144. Dead cells were excluded by DAPI staining (Invitrogen). Cells were sorted on a BD Influx into 1 mL 1× IMDM + 50% FBS in a 1.5 mL LoBind tube.

E14.5 FL HSCs: B6C3F1/J female mice were mated with B6129SF1/J male mice, and fetal livers were dissected from E14.5 embryos. Single cell suspensions were prepared by mechanical dissociation and expelling the cells through 40 μm cell strainer, followed by red blood cell lysis using ACK lysing buffer (Lonza). Cells were washed and stained with an antibody cocktail of CD150, CD117 (c-Kit), Ly-6A/E (Sca1), CD48, Ly6G/Ly-6C (Gr-1), CD11b, TER-119, CD4, CD8a, CD45R/B220, CD3ε and CD11c. Washed cells were filtered through 40 µm cell strainer twice and Hoechest 33258 stock solution was added just prior to FACS. Cells were sorted by yield sort first followed by purity sort to obtain long-term HSCs (Lin^-^ Sca1^+^c-Kit^+^ CD150^+^ CD48^-^).

Bone marrow HSCs: Cells from long bones were suspended and incubated with anti-CD16/32 and anti-CD117 microbeads (Miltenyi Biotec) sequentially. Cells were washed twice with 1× PBS + 2% FBS and fed to autoMACS (Miltenyi Biotec) to enrich for CD117^+^ cells. Enriched cells were stained with the antibody cocktail against the following antigens: CD150, CD117 (c-Kit), Ly-6A/E (Sca1), CD135, CD48, Ly6G/Ly-6C (Gr-1), CD11b, TER-119, CD4, CD8a, CD45R/B220, CD3ε and CD11c (Supplemental Table S13) at 4 °C for 15 min in the dark. Washed cells were filtered twice through a 40 µm cell strainer and Hoechest 33258 stock solution was added just prior to sorting. Cells were sorted by yield sort first followed by purity sort to obtain long-term BM HSCs (Lin^-^Sca1^+^c-Kit^+^CD150^+^CD48^-^CD135^-^).

E10.5 endothelial cells for limiting dilution assay and scRNA-Seq: Embryos were collected from *Sp3*^+/-^ females mated to *Sp3*^+/-^ males. The yolk sac was removed for genotyping, and the embryo was dissected in 1× PBS + 10% FBS (Gibco) + 1% Penicillin-Streptomycin (Gibco). Dorsal aorta regions were isolated by dissection and dissociated in 0.125% collagenase (Sigma) for 40 min. Cells were washed and stained with the following antibodies (1:200) for the limiting dilution assay sort: CD45, Ter119, ESAM, CD41, CD144, CD31, CD44, and DAPI (Invitrogen, D3571) for viability. The following antibodies (1:200) were used for scRNA-Seq sort: Ter119, ESAM, CD41, CD144, CD31, and DAPI (Invitrogen, D3571) for viability. The cells were pooled by genotype and sorted on a BD Influx into αMEM (Thermo Scientific) for limiting dilution assay and 1× PBS + 10% FBS for scRNA-Seq.

**Zebrafish maintenance**

Zebrafish were maintained according to the IACUC-approved protocols at Children’s Hospital of Philadelphia. Zebrafish were bred, and embryos were collected for microinjection within 45 min post fertilization. Post microinjection, embryos were raised in E3 medium in 100 mm Petri dishes at a density of fewer than 100 embryos per dish. All larvae were incubated at 25 °C.

**Limiting dilution assay**

OP9 stromal cells were cultured in αMEM (Thermo Scientific) containing 10% FBS (Gibco) and 1% Penicillin-Streptomycin (Gibco). One day prior to the initiation of the HE assay the OP9 cells were plated at a concentration of 4000 cells/well in 96-well plates. Hemogenic endothelial cell limiting dilution assays were performed by culturing sorted populations on OP9 cells supplemented with 20% FBS + 1% Penicillin-Streptomycin and 10 ng/mL each of SCF, IL-3, Flt3L and IL-7 (all cytokines from PeproTech). Six dilutions were performed with 3 replicates of each. On days 7-10, wells were inspected for hematopoietic colonies using a light microscope. Samples were analyzed using a BD LSRII flow cytometer using the following antibodies (1:200): CD45, c-KIT, Ter119, CD41, and Aqua LIVE/DEAD (Invitrogen, L34966) for viability. Positive wells were identified as wells containing CD41^+^, CD45^+^ and/or Ter119^+^ cells. The HE frequency was calculated using ELDA software [(Hu and Smyth 2009)](https://paperpile.com/c/POiN9L/vo6x).

**Colony forming progenitor assay**

For SP3 colony forming assays, whole yolk sac was dissociated in 0.125% collagenase (Sigma) and 0.03 embryonic equivalents of yolk sacs were plated in replicates. Colonies were scored on a light microscope ten days after plating.

**ChIP-qPCR**

After the ChIP step in the Low-Cell-Number ChIP-Seq protocol, purified DNA from matched IP sample and input sample were subjected to qPCR analysis using iQ SYBR Green Supermix (Bio-Rad). The fold enrichment was computed as 2^-ΔΔCt^, where ΔΔCt is the difference in ΔCt values between the IP sample and the input sample. ΔCt is the cycle number difference between a target region (well-known or predicted enhancer) and a negative control region (without any histone modification signal). Primer sequences are included in Supplemental Table S14.

**ChIP-Seq data processing**

Sequencing reads were mapped to the mouse genome (mm9) using Bowtie2 (v2.2.2) with default parameter setting [(Langmead et al. 2009)](https://paperpile.com/c/POiN9L/7anL). Only uniquely mapped reads with fewer than 2 mismatches were used to compute a normalized signal for each 200 bp bin across the genome. Normalized signal is defined as the following: $normalized signal=IP\left( \frac{Reads in each bin}{Total uniquely mapped reads}\times1000000 \right)-Input\left( \frac{Reads in each bin}{Total uniquely mapped reads}\times1000000 \right)$.

To assess the reproducibility of ChIP-Seq data, for a given histone modification mark, we divided the mouse genome into consecutive 1 kb windows. Normalized ChIP-Seq signals in windows were used for computing genome-wide correlation using Pearson correlation coefficient. Windows with zero signals in both replicates were removed before calculating correlation.

**RNA-Seq data processing and differential expression analysis**

Sequencing reads were aligned to the mouse genome (mm9) using Tophat [(Kim et al. 2013)](https://paperpile.com/c/POiN9L/dXtY). Only uniquely mapped reads with fewer than 2 mismatches were used for downstream analyses. The Ensembl database (release 66) was used as the source of gene annotation. The featureCounts function of the Subread package [(Liao et al. 2014)](https://paperpile.com/c/POiN9L/tSu3) was used to extract both gene-level and transcript-level read counts. Differential expression analysis was performed using the edgeR algorithm [(Robinson and Oshlack 2010)](https://paperpile.com/c/POiN9L/w8nc). ANOVA was first used to identify genes that are differentially expressed across any of the five populations with a False Discovery Rate (FDR) < 0.01. Next, pairwise comparisons were conducted to identify genes differentially expressed between two populations with FDR < 0.05 and fold change > 2.

**Clustering of expression profile using STEM**

Gene expression values were expressed as Fragment Per Kilobase of transcript per Million mapped reads (FPKM). Short-time series expression miner (STEM) [(Ernst et al. 2005)](https://paperpile.com/c/POiN9L/KoFGj) was used for clustering with default parameter setting except that minimum correlation of profile was set to 0.9 to group similar expression profiles.

**Enhancer prediction using histone modification ChIP-Seq data**

Enhancers were predicted using the CSI-ANN algorithm [(Firpi et al. 2010)](https://paperpile.com/c/POiN9L/lzPKn). The inputs to the algorithm are normalized ChIP-Seq signals of four histone marks (H3K4me1, H3K4me3, H3K27ac, and H3K27me3). The algorithm combines signals of all histone marks and uses an artificial neural network-based classifier to make predictions.

**Identification of enriched TF binding motifs at enhancers**

TF DNA binding motifs were downloaded from the Cis-BP database [(Weirauch et al. 2014)](https://paperpile.com/c/POiN9L/AN8H). Similar motifs were merged using TomTom [(Bailey et al. 2009)](https://paperpile.com/c/POiN9L/b6mr). Find Individual Motif Occurrences (FIMO) [(Grant et al. 2011)](https://paperpile.com/c/POiN9L/DuPO) was used to scan enhancer sequences defined by CSI-ANN. A *P*-value cutoff of 1E-5 (multiple testing corrected using Bonferroni’s method) was used to identify binding sites according to the TF motif model. For background, we used 9,500 enhancers from 19 non-hematopoietic cell lines reported by the ENCODE project [(Shen et al. 2012)](https://paperpile.com/c/POiN9L/oexA) (brown adipose tissue, brain (E14.5), cerebellum, cortex, heart (E14.5), heart, intestine, kidney, limb (E14.5), liver (E14.5), liver, lung, mouse embryonic fibroblasts, mouse ESC, olfactory, placenta, spleen, testes, and thymus, Supplemental Table S15). Hypergeometric distribution was used to compute the enrichment *P*-values. Benjamini-Hochberg method was used to correct *P*-values for multiple testing.

**Construction of transcriptional regulatory networks**

We developed a computational pipeline to infer target genes of a TF based on the probabilities of enhancer, TF binding site, and enhancer-promoter linkage. The probability of an enhancer based on its histone mark signals, *P_enh_*, is defined by the CSI-ANN algorithm. The probability of an enhancer-promoter linkage, *P_EP_*, is defined by the IM-PET algorithm. To determine if a TF occupies an enhancer, we first use a compendium of 1147 mouse TF motifs curated in the Cis-BP database [(Weirauch et al. 2014)](https://paperpile.com/c/POiN9L/AN8H). We calculate the probability that a TF is bound to its site in the enhancer, *P_TFBS_*. Given an enhancer sequence *l* and a PSSM *M* representing a TF binding motif, the binding probability can be approximated as following according to [(Stormo and Fields 1998)](https://paperpile.com/c/POiN9L/ZpI5h):

$P\left( A,M \right)=\frac{\sum_{j=1}^{l} e^{s_{j}}}{\sum_{j=1}^{g} e^{s_{j}}}$where *l* is the length of *A* and *g* is the length of the background sequence, *S_j_* is the score of the sequence word starting at position *j* according to the PSSM. In our analysis, we will use the entire sequence of chromosome one as the background. Finally, the probability of a TF regulating a target gene, *P_TG_*, is defined to be the product of the three probabilities, *P_TG_* = *P_enh_* × *P_TFBS_* × *P_EP_*. We included the edges in the network having probability greater than the optimal threshold computed for the AUC for benchmark dataset in our earlier study [(Gao et al. 2019)](https://paperpile.com/c/POiN9L/nzBqh). The network was further pruned by motif analysis such that TFs not having a motif with a significant motif hit in an enhancer targeting the designated target were pruned.

**Identification of key transcription factors based on their regulatory potential**

We used the constructed TRNs to identify key transcriptional factors for the EHT. To this end, we assume that key TFs are closer to the set of differentially expressed genes in the TRN, either via direct or indirect connections. Based on this assumption, we computed a distance between two genes, *i* and *j*, in the TRN as following:

$$d_{ij}=1-\frac{{log}_{p_{i}}+{log}_{p_{j}}}{2{log}_{p_{min}}}$$

where *p_i_* and *p_j_* are the differential expression *P*-values for genes *i* and *j*, respectively. *p_min_* is the minimum differential expression *P*-value among all genes in the TRN. With the distance-weighted TRNs, we calculated an average shortest distance between a given TF and all differentially expressed genes in the network. Statistical significance of average shortest distance was computed using a null distribution based on the given TF and randomly selected genes. We selected the sets of key TF as follows. First we ranked the TFs based on their distance to differentially expressed genes in the TRN. Then, starting from the highest ranked TF, we iteratively computed the cumulative union of the regulons of TF sets, having *n* number of TFs, at *n*^th^ step. Next, we computed the difference between the expected regulon size with the actual regulon size. We set the cutoff for the number of key TFs at the inflection point of the curve.

**Identification of mouse orthologs in zebrafish**

Orthologs of *Sp3* and *Maz* in zebrafish were identified using four databases: MGI (http://www.informatics.jax.org), ZFIN (https://zfin.org), OrthoRetriever (http://lighthouse.ucsf.edu/orthoretriever) and DIOPT (http://www.flyrnai.org/diopt).

**CRISPR-Cas9 mediated gene knockout in zebrafish.**

Genes were knocked out with Alt-R™ CRISPR-Cas9 crRNA from Integrated DNA Technologies (IDT) following the manufacturer’s instructions. Briefly, crRNAs were designed using CRISPOR (http://crispor.tefor.net/). To generate mutants, 900 picoliter of 7.5 µM ribonucleoprotein complex (7.5 µM crRNA and 7.5 µM Cas9 enzyme (IDT)) was injected directly into 1-cell-stage embryos (strain Casper) and up to 200 zebrafish embryos were injected per target gene. Editing efficiency was measured using the TIDE assay. The mRNA and protein levels of target genes were measured using RT-qPCR and Western Blot, respectively. Primer sequences are included in Supplemental Table S14.

**Tracking of indels by decomposition (TIDE)**

Genomic DNA was extracted from embryos using the HotSHOT method [(Meeker et al. 2007)](https://paperpile.com/c/POiN9L/VeAA). A short stretch of genomic region (∼600-800 bp) ﬂanking the target site was ampliﬁed using Q5 Hot Start High Fidelity 2 × Master Mix (NEB). Amplified sequences were purified with the QIAquick PCR purification kit (Qiagen) and sequenced by Sanger sequencing. A fragment amplified using wild type zebrafish was used as a control. Primer sequences are included in Supplemental Table S14.

**Dissection of zebrafish embryos and flow cytometry**

Embryos were collected and anaesthetized in E3 medium containing 0.1 mg/mL tricaine (Sigma). Disaggregation into single-cell suspension was performed as described previously (Stachura and Traver 2016). Flow cytometry was performed on a CytoFLEX S (Beckman Coulter). Data analyses were performed using FlowJo software (FlowJo LLC).

**Analysis of blood vessel diameter in zebrafish**

Blood vessel diameter analysis was done using 48 hpf zebrafish embryos. For dorsal aorta (DA) and posterior cardinal vein (PCV), measurements were taken at the midway point between intersegmental vessels (ISV) along the yolk extension, and the mean was used as an average diameter per embryo. For ISV, 4 measurements were made along each ISV between the DA and the dorsal longitudinal anastomotic vessel (DLAV), and the mean was used as an average diameter per ISV.

**Whole-mount in situ hybridization (WISH) in zebrafish**

Zebrafish embryos were dechorionated using pronase, fixed in 4% paraformaldehyde at 4 °C overnight, dehydrated in methanol and stored at -20 °C. Both sense and anti-sense RNA probes were synthesized from restriction enzyme linearized plasmids transcribed with T7 or SP6 polymerase in the presence of RNA DIG labelling mix (Roche). Newly synthesized probes were purified using mini QuickSpin columns according to the manufacturer’s protocol (Roche). Synthesis of RNA probes was performed as described [(North et al. 2007; Song et al. 2004)](https://paperpile.com/c/POiN9L/KXAX+YZo9). WISH was performed using an established protocol [(Thisse and Thisse 2008)](https://paperpile.com/c/POiN9L/E5HP) with some modifications. Briefly, embryos stored in methanol were rehydrated in successive dilutions of methanol in 1× PBS. After washing in 1× PBST, embryos were permeabilized by digestion with 10 µg/mL proteinase K at room temperature. Digestion with proteinase K was stopped by 4% paraformaldehyde in 1× PBS and embryos were washed in 1× PBST. After prehybridization at 65 °C, the embryos were hybridized in a 70 °C hybridization oven overnight. Following several washes, embryos were incubated with anti-DIG antibody at 4 °C overnight. Embryos were then washed, stained and mounted in 100% glycerol for imaging using a Leica S8 APO stereo microscope with a Leica DFC420 camera. At least 20 embryos per condition were scored.

**Whole-mount immunofluorescence and confocal microscopy**

Embryos and yolk sacs were prepared as described previously [(Yokomizo et al. 2012)](https://paperpile.com/c/POiN9L/tECe). The following primary antibodies were used: CD117 (1:250), CD31 (1:500), RUNX1 (1:250), and SOX17 (1:250). Secondary antibodies were Alexa Fluor 647 (1:500), Alexa Fluor 555 (1:1000) and Alexa Fluor 488 (1:1000). Images were acquired on a Zeiss LSM 710 AxioObserver inverted microscope with ZEN 2011 software. The Zeiss LSM 710 is equipped with 488, 543 and 633 nm wavelengths. Images were processed with Fiji software [(Schindelin et al. 2012)](https://paperpile.com/c/POiN9L/Oldz).

**Quantification of confocal images**

Single RUNX1^+^c-KIT^lo/-^ CD31^+^ cells in the wall of the dorsal aorta were counted for HE cells, and single RUNX1^+^c-KIT^+^CD31^+^ cells in the lumen of the dorsal aorta were counted for intra-aortic cluster cells. Cells were counted within a measured region centered on the vitelline artery using Fiji software [(Schindelin et al. 2012)](https://paperpile.com/c/POiN9L/Oldz). Raw counts of the number of cells were divided by the measured region. For quantification of the diameter of yolk sac vessels, a random region of 62,500µm^2^ was selected from images of small vessels of the yolk sac and 150 vessels were measured within the region. Vessels larger than 40µm were not measured.

**scRNA-Seq**

Sorted cells were immediately processed for library preparation using the 10× Genomics Chromium controller, in conjunction with the Chromium Single Cell 3′ Reagent Kit v3, according to manufacturer’s protocol (CG00052, Rev D). Libraries were quantified using the dsDNA High-Sensitivity (HS) Assay Kit (Invitrogen) on the Qubit ﬂuorometer and the qPCR-based KAPA assay (Kapa Biosystems). Library quality assessment was performed on the Agilent 2100 Bioanalyzer in combination with the Agilent High Sensitivity DNA kit. Indexed libraries were pooled and sequenced on an NextSeq 550 using paired-end 26 × 98 bp read length.

##### **scRNA-Seq data processing and filtering**

Sequencing reads were first pre-processed with the 10× Genomics Cell Ranger pipeline and aligned to the mouse mm10 reference genome. An initial filtering was performed on the raw gene-barcode matrix outputted by the cellranger count function, removing barcodes that have fewer than 1,000 transcripts (quantified by unique molecular identifier (UMI)) and 1,000 expressed genes (“expressed” means that there is at least 1 transcript from the gene in the cell). Barcodes that pass this filter were considered as cells and were used for downstream analysis.

##### **UMAP projection and cell type assignment**

To confidently assign cell types, we projected our scRNA-Seq data onto a UMAP constructed with a reference scRNA-Seq dataset from Zhu et al (Zhu et al. 2020). We first removed contaminant cell types such as erythroid progenitors from our data based on an initial clustering and differential expression analysis. The rest of the cells were then projected onto the EHT trajectory shown in Figure 2B from Zhu *et al*. using the following procedure:

(1) Re-compute principle components (PCs) on log-transformed expression matrix from Zhu *et al*. using variably expressed genes, defined as the top 20% of genes ranked by the Gini coefficient across cell types;

(2) Use the top 20 PCs to compute a UMAP using the *umap* function from uwot R package (“cosine” distance metric, 10 nearest neighbors, and default for the rest of the parameters);

(3) Using the PCA loading matrix, project the SP3 wild type and null cell data onto the PCA space computed in (1), then predict UMAP embedding using *umap_transform* function with the previously computed UMAP model. The final co-embedding of our data with Zhu *et al*. data are shown in Figure 7G.

(4) Cells from this study were then annotated with a 3-nearest-neighbor classifier built on the umap co-embedding. For example, if a cell from this study has 2 out of 3 nearest neighbors from Zhu *et al*. identified as HE, then it was assigned as an HE cell. Equal votes were assigned randomly.

##### **Proportion test on cell type transition**

For consecutive cell types along the EHT trajectory, we tested if the proportion of the later-stage cell type is significantly lower in SP3 null versus wild type samples. For example, to test if the Wnt^high/low^ AE to Conflux AE transition was significantly reduced in SP3 null samples, we compared the ratio of Conflux AE cell counts to the sum of Wnt^low^ and Wnt^high^ AE cell counts in null versus wild type samples, using the two-sample proportion test (implemented in the *prop.test* function in R), with alternative hypothesis set to “less”.

**Reagents**

All antibodies used in this study are listed in Supplemental Table 13. All PCR primers are listed in Supplemental Table 14.

**Supplemental Figures**

**Supplemental Figure S1. FACS gating strategy for purification of the five studied populations.**

1. Endothelial cells (CD31^+^ Kit^-^ CD45^-^ CD41^-^ Runx1:GFP^-^) and hemogenic endothelial cells (CD31^+^ Kit^lo^ CD45^-^ CD41^-^ Runx1:GFP^+^) were purified from the caudal region of E10.5 Runx1:GFP mouse embryos. The vitelline and umbilical arteries were severed from the yolk sac and placenta, respectively, and included in the samples. The liver, digestive tube, tail and limb buds were removed. Shown are representative FACS plots depicting the gating strategy. Numbers on the x- and y-axes are indicated on the first plot on the left, and unless changed are not depicted on plots to the right of the preceding plot.
2. Representative FACS plots for isolation of pre-HSCs (CD31^+^ CD144^+^ ESAM^+^ c-Kit^+^ Ly6a:GFP^+^) from E11.5 Ly6a:GFP embryos.
3. Representative FACS plots from purification of E14.5 FL HSCs (CD150^+^CD48^-^Lineage^-^Sca1^+^c-Kit^+^).
4. Representative FACS plots from purification of BM HSCs (CD150^+^CD48^-^CD135^-^Lineage^-^Sca1^+^c**-**Kit^+^).

**Supplemental Figure S2. Reproducibility of RNA-Seq data.** Pearson correlation coefficient of gene expression level of two biological replicates.

**Supplement Figure S3. RNA-seq differential expression analysis between adjacent stages.**

(**A**) Enriched GO biological process terms among expression clusters. Color shade is proportional to the minus logarithm of enrichment *P*-value. GO: Gene Ontology.

(**B**) Top GO terms for genes downregulated in HE relative to Endo. (**C**) Top GO terms for genes downregulated in pre-HSCs relative to HE. (**D**) Volcano plot of DEGs between pre-HSCs and FL HSCs; selected genes are listed. (**E**) Left, top GO terms for genes down regulated in FL HSCs relative to pre-HSCs. Right, Top GO terms for genes up regulated in FL HSCs relative to pre-HSCs. (**F**) Volcano plot of DEGs between FL HSCs and BM HSCs; selected genes are listed. (**G**) Left, top GO terms for genes down regulated in BM HSCs relative to FL HSCs. Right, Top GO terms for genes up regulated in BM HSCs relative to FL HSCs.

**Supplemental Figure S4. Mapping statistic of histone mark ChIP-Seq data.** ChIP-Seq reads were mapped to the mouse genome (assembly mm9) using Bowtie2 with default parameters. Light blue bars show the number of total reads (in millions). Red dots show the percentages of uniquely mapped reads.

**Supplemental Figure S5. Reproducibility of ChIP-Seq data.** Shown are scatter plots of normalized ChIP-Seq signals of biological replicate samples. ChIP-Seq signals were normalized as (IP_window_/IP_total_) - (Input_window_/Input_total_). Normalized signals were multiplied by 10 million for display purpose. “IP” and “Input” denote immunoprecipitated and input samples; “window” and “total” denote numbers of sequencing reads in a 200 bp window and total number of reads in an experiment. **“**r**”** denotes Pearson correlation coefficient. ChIP-Seq signals in consecutive 1 kb windows (windows for 0 signals in both duplicates were removed) across the genome were used for computing correlations.

**Supplemental Figure S6. Validation of histone modification ChIP-Seq peaks.** All cell populations were purified using FACS. ChIP was performed on each cell population using anti-H3K4me1, H3K4me3, H3K27me3, or H3K27ac antibodies. ChIP enrichment was determined using qPCR. Two independent experiments were performed and each sample was measured in duplicates. (**A-D)**, endothelium; (**E-H)**, hemogenic endothelium; (**I-L)**, pre-HSC; (**M-P)**, FL HSC; (**Q-T)**, BM HSC. Dashed line indicates two fold enrichment.

**Supplemental Figure S7. Characteristics of predicted enhancer-promoter pairs.**

Cumulative distributions of (**A)** the number of promoters that contact an enhancer

and (**B)** the number of enhancers that contact a promoter and (**C)** the number of intervening promoters between a linked enhancer and promoter at the five developmental stages. (**D)** Upset diagram of predicted enhancer-promoter pairs at the five developmental stages. Enhancers were predicted with 5% FDR cutoff using the CSI-ANN algorithm. EP pairs were predicted using the IM-PET algorithm. Each vertical bar represents the number of overlapped EP pairs among the given enhancer sets (indicated by the series of connected solid circles below x-axis). Number of EP pairs in each cell type is indicated by horizontal bars at the bottom.

**Supplemental Figure S8. Determination of threshold for key regulator identification during each developmental transition.** Blue line represents the cumulative percentage of target genes of the set of identified regulators, starting from the highest ranked TF to the lowest ranked TF. Dotted black line represents the expected coverage of target genes if all TFs have target gene sets of the same size. Purple line represents the difference between the observed coverage vs expected coverage. x-axis, rank order of predicted TFs. Red vertical line represents the determined threshold based on the maximal difference between the observed and expected target gene coverage. **(A)** Endo to HE transition. **(B)** HE to pre-HSC transition. **(C)** pre-HSC to FL HSC transition. **(D)** FL HSC to BM HSC transition.

**Supplemental Figure S9. Expression levels of predicted key TFs across the five developmental stages.**

**Supplemental Figure S10. Expression of *runx1* and *c-myb* at different time points in wild type zebrafish embryos.** Representative whole-mount in situ hybridization (WISH) images of expression of *runx1* and *c-myb* at different time points. *runx1* (left column) and *c-myb* (right column) mRNA levels were examined at 4 time points (24 hpf, 27 hpf, 30 hpf and 33 hpf for *runx1*; 27 hpf, 30 hpf, 33 hpf and 36 hpf for *c-myb*). n = 3 to 7 embryos per time point for both *runx1* and *c-myb*.

**Supplemental Figure S11. *Sp3* and *Maz* knockout zebrafish have blood vessel development. (A)** Schematic of the experiment and diagram of vasculature of zebrafish. **(B)** Representative confocal images of the blood vessels in wild type, *Sp3* and *Maz* orthologs knockout embryos. The blood vessels are visualized by using a transgenic zebrafish line Tg(kdrl:mCherry) in which blood vessels are marked with red fluorescence. Scale bar, 200 µm. **(C)** Quantification of average vessel diameter in wild type, *Sp3*, and *Maz* orthologs knockout embryos. DA, dorsal aorta (n = 5); PCA, posterior cardinal vein (n = 5); ISV, intersegmental blood vessels (n = 40). *P*-values were based on unpaired Student's *t*-test. n = 5 embryos for each condition. NS, not significant; error bars indicate standard error of the mean (SEM).

**Supplemental Figure S12. Characterization of loss of SP3 on the vasculature in mouse embryos. (A)** Confocal z-projections (z-intervals = 2.5µm) of yolk sacs from E10.5 *Sp3^+/+^* and *Sp3^-/-^* mouse embryos showing large vessels (top panel) and small vessels (bottom panel). Yolk sacs were immunostained for CD31 and the arterial marker SOX17. Scale bars: 100 µm. **(B)** An example region of the yolk sac (top left) and a zoom in on this region (top right) showing where the diameters of the vessels were measured, indicated with white lines. Bottom panel shows quantification of the diameter of 150 small vessels (< 40 µm) measured from a random 62,500 µm^2^ region of small vessels (mean ± SD, two-way ANOVA of grouped data, no significant difference between genotypes in each bin of vessel size). Data are from 3 embryos of each genotype from 3 independent litters. **(C)** Expression of several example genes that are known to be regulated by flow (*Nos3, Cav1, Klf2,* and *Nrf2*) in all endothelial cells. Expression data are from scRNA-Seq of endothelial cells (Ter119^-^CD4^lo/-^CD31^+^VEC^+^ESAM^+^) purified from the dorsal aortaes of E10.5 *Sp3^+/+^* or *Sp3^-/-^* embryos. Each point represents a single cell.

**Supplemental Tables**

**Supplemental Table 1. Description of purified cells for RNA-Seq.**

**Supplemental Table 2. Description of purified cells for ChIP-Seq..**

**Supplemental Table 3. List of differentially expressed genes at each developmental stage.**

**Supplemental Table 4. Summary statistics of ChIP-Seq data generated in this study.**

**Supplemental Table 5. Known mouse hematopoietic enhancers.**

**Supplemental Table 6. Overlapped enhancers with iChIP.**

**Supplemental Table 7. List of enhancer-promoter pairs.**

**Supplemental Table 8. List of primed enhancers.**

**Supplemental Table 9. List of enhancers that change epigenetic state between primed and activate enhancers during HSC ontogeny.**

**Supplemental Table 10. List of TF-target pairs in constructed transcriptional regulatory networks for four developmental transitions.**

**Supplemental Table 11. Sequences of guide RNAs for CRISPR-Cas9-mediated gene knockout in zebrafish.**

**Supplemental Table 12. CRISPR editing efficiency of *sp3 and maz homologs.*** For each homolog pair (*sp3a* + *sp3b*, *maza* + *si:ch211-166g5.4*), up to 10 individual embryos were assessed for the editing efficiency using the Tracking of Indels by Decomposition (TIDE) assay. Values are mean editing efficiencies of the 10 individual embryos.

**Supplemental Table 13. Antibodies used in this study.**

**Supplemental Table 14. List of PCR primers.**

**Supplemental Table 15. List of ENCODE enhancers for TF enrichment analysis.**

**Supplemental Table 16. Number of cells in each population from *Sp3* scRNA-seq.**

**References**

[Bailey TL, Boden M, Buske FA, Frith M, Grant CE, Clementi L, Ren J, Li WW, Noble WS. 2009. MEME SUITE: tools for motif discovery and searching. *Nucleic Acids Res* **37**: W202–8.](http://paperpile.com/b/POiN9L/b6mr)

[Ernst J, Nau GJ, Bar-Joseph Z. 2005. Clustering short time series gene expression data. *Bioinformatics* **21 Suppl 1**: i159–68.](http://paperpile.com/b/POiN9L/KoFGj)

[Firpi HA, Ucar D, Tan K. 2010. Discover regulatory DNA elements using chromatin signatures and artificial neural network. *Bioinformatics* **26**: 1579–1586.](http://paperpile.com/b/POiN9L/lzPKn)

[Gao P, Uzun Y, He B, Salamati SE, Coffey JKM, Tsalikian E, Tan K. 2019. Risk variants disrupting enhancers of TH1 and TREG cells in type 1 diabetes. *Proc Natl Acad Sci U S A* **116**: 7581–7590.](http://paperpile.com/b/POiN9L/nzBqh)

[Grant CE, Bailey TL, Noble WS. 2011. FIMO: scanning for occurrences of a given motif. *Bioinformatics* **27**: 1017–1018.](http://paperpile.com/b/POiN9L/DuPO)

[Hu Y, Smyth GK. 2009. ELDA: extreme limiting dilution analysis for comparing depleted and enriched populations in stem cell and other assays. *J Immunol Methods* **347**: 70–78.](http://paperpile.com/b/POiN9L/vo6x)

[Kim D, Pertea G, Trapnell C, Pimentel H, Kelley R, Salzberg SL. 2013. TopHat2: accurate alignment of transcriptomes in the presence of insertions, deletions and gene fusions. *Genome Biol* **14**: R36.](http://paperpile.com/b/POiN9L/dXtY)

[Langmead B, Trapnell C, Pop M, Salzberg SL. 2009. Ultrafast and memory-efficient alignment of short DNA sequences to the human genome. *Genome Biol* **10**: R25.](http://paperpile.com/b/POiN9L/7anL)

[Liao Y, Smyth GK, Shi W. 2014. featureCounts: an efficient general purpose program for assigning sequence reads to genomic features. *Bioinformatics* **30**: 923–930.](http://paperpile.com/b/POiN9L/tSu3)

[Ma X, Robin C, Ottersbach K, Dzierzak E. 2002. The Ly-6A (Sca-1) GFP transgene is expressed in all adult mouse hematopoietic stem cells. *Stem Cells* **20**: 514–521.](http://paperpile.com/b/POiN9L/D4NL)

[Meeker ND, Hutchinson SA, Ho L, Trede NS. 2007. Method for isolation of PCR-ready genomic DNA from zebrafish tissues. *Biotechniques* **43**: 610, 612, 614.](http://paperpile.com/b/POiN9L/VeAA)

[North TE, Goessling W, Walkley CR, Lengerke C, Kopani KR, Lord AM, Weber GJ, Bowman TV, Jang IH, Grosser T, et al. 2007. Prostaglandin E2 regulates vertebrate haematopoietic stem cell homeostasis. *Nature* **447**: 1007–1011.](http://paperpile.com/b/POiN9L/KXAX)

[Robinson MD, Oshlack A. 2010. A scaling normalization method for differential expression analysis of RNA-seq data. *Genome Biol* **11**: R25.](http://paperpile.com/b/POiN9L/w8nc)

[Schindelin J, Arganda-Carreras I, Frise E, Kaynig V, Longair M, Pietzsch T, Preibisch S, Rueden C, Saalfeld S, Schmid B, et al. 2012. Fiji: an open-source platform for biological-image analysis. *Nat Methods* **9**: 676–682.](http://paperpile.com/b/POiN9L/Oldz)

[Shen Y, Yue F, McCleary DF, Ye Z, Edsall L, Kuan S, Wagner U, Dixon J, Lee L, Lobanenkov VV, et al. 2012. A map of the cis-regulatory sequences in the mouse genome. *Nature* **488**: 116–120.](http://paperpile.com/b/POiN9L/oexA)

[Song HD, Sun XJ, Deng M, Zhang GW, Zhou Y, Wu XY, Sheng Y, Chen Y, Ruan Z, Jiang CL, et al. 2004. Hematopoietic gene expression profile in zebrafish kidney marrow. *Proc Natl Acad Sci U S A* **101**: 16240–16245.](http://paperpile.com/b/POiN9L/YZo9)

[Stormo GD, Fields DS. 1998. Specificity, free energy and information content in protein-DNA interactions. *Trends Biochem Sci* **23**: 109–113.](http://paperpile.com/b/POiN9L/ZpI5h)

[Thisse C, Thisse B. 2008. High-resolution in situ hybridization to whole-mount zebrafish embryos. *Nat Protoc* **3**: 59–69.](http://paperpile.com/b/POiN9L/E5HP)

[Weirauch MT, Yang A, Albu M, Cote AG, Montenegro-Montero A, Drewe P, Najafabadi HS, Lambert SA, Mann I, Cook K, et al. 2014. Determination and inference of eukaryotic transcription factor sequence specificity. *Cell* **158**: 1431–1443.](http://paperpile.com/b/POiN9L/AN8H)

[Yokomizo T, Yamada-Inagawa T, Yzaguirre AD, Chen MJ, Speck NA, Dzierzak E. 2012. Whole-mount three-dimensional imaging of internally localized immunostained cells within mouse embryos. *Nat Protoc* **7**: 421–431.](http://paperpile.com/b/POiN9L/tECe)

Zhu Q, Gao P, Tober J, Bennett L, Chen C, Uzun Y, Li Y, Howell ED, Mumau M, Yu W et al. 2020. Developmental trajectory of pre-hematopoietic stem cell formation from endothelium. *Blood*.
