# Supplementary material for: Transcriptional regulatory network controlling the ontogeny of hematopoietic stem cells
Source: Genes Dev. 2020 Jul 1;34(13-14):950–64. doi: 10.1101/gad.338202.120 (PMC7328518; doi:10.1101/gad.338202.120)
Supplement: Supplemental Material [file supp_gad.338202.120_Supplemental_Table_S1-NEW.docx]

**Supplemental Table 1. Description of purified cells used for RNA-Seq.**

| Cell type | Cell surface phenotype | Developmental Stage | No. of biological replicates | No. of embryos/mice dissected | No. of cells per assay |
| --- | --- | --- | --- | --- | --- |
| BM HSC | Lineage^-^Sca-1^+^c-Kit^+^  CD150^+^CD34^-^ | 6-8 weeks | 2 | 10 | 38109 |
| FL HSC | Lineage^-^Sca-1^+^c-Kit^+^  CD150^+^CD48^-^ | E14.5 | 2 | 28 | 42104 |
| Pre-HSC | CD31^+^CD144^+^ESAM^+^  c-Kit^+^Ly6a:GFP^+^ | E11.5 | 3 | 122 | 17820 |
| HE | CD31^+^Runx1:GFP^+^  c-Kit^lo/neg^CD45^-^CD41^-^ | E10.5 | 2 | 264 | 22851 |
| Endo | CD31^+^Runx1:GFP^-^  c-Kit^-^CD45^-^CD41^-^ | E10.5 | 2 | 264 | 294902 |
