# Supplementary material for: Transcriptional regulatory network controlling the ontogeny of hematopoietic stem cells
Source: Genes Dev. 2020 Jul 1;34(13-14):950–64. doi: 10.1101/gad.338202.120 (PMC7328518; doi:10.1101/gad.338202.120)
Supplement: Supplemental Material [file supp_gad.338202.120_Supplemental_Table_S2-NEW.docx]

**Supplemental Table S2. Description of purified cells for ChIP-Seq.**

| Cell type | Cell surface phenotype | Developmental Stage | No. of biological replicates | No. of embryos/mice dissected | No. of cells per assay |
| --- | --- | --- | --- | --- | --- |
| BM HSC | Lineage^-^Sca-1^+^c-Kit^+^  CD150^+^CD34^-^ | 6-8 weeks | 2 | 26 | 18808 |
| FL HSC | Lineage^-^Sca-1^+^c-Kit^+^  CD150^+^CD48^-^ | E14.5 | 2 | 216 | 19823 |
| Pre-HSC | CD31^+^CD144^+^ ESAM^+^  c-Kit^+^ Ly6a:GFP^+^ | E11.5 | 2 | 242 | 26160 |
| HE | CD31^+^Runx1;GFP^+^  c-Kit^lo/neg^CD45^-^CD41^-^ | E10.5 | 2 | 542 | 19647 |
| Endo | CD31^+^Runx1:GFP^-^  c-Kit^-^CD45^-^CD41^-^ | E10.5 | 2 | 44 | 21680 |
