# Supplementary material for: Transcriptional regulatory network controlling the ontogeny of hematopoietic stem cells
Source: Genes Dev. 2020 Jul 1;34(13-14):950–64. doi: 10.1101/gad.338202.120 (PMC7328518; doi:10.1101/gad.338202.120)
Supplement: Supplemental Material [file supp_gad.338202.120_Supplemental_Table_S4.docx]

**Supplemental Table S4. Summary statistics of ChIP-Seq data generated in this study.**

| Cell type | Marker | Replicate | Total reads (Million) | % mapped reads | % uniquely mapped reads |
| --- | --- | --- | --- | --- | --- |
| BM HSC | H3K4me1 | 1 | 26.4 | 90.5 | 68.5 |
|  |  | 2 | 28.8 | 91.7 | 69.7 |
|  | H3K4me3 | 1 | 37.7 | 88 | 65.6 |
|  |  | 2 | 29.8 | 88.9 | 65.6 |
|  | H3K27ac | 1 | 30.1 | 90.3 | 67.1 |
|  |  | 2 | 35.7 | 89.8 | 66.6 |
|  | H3K27me3 | 1 | 28.1 | 88.9 | 59.4 |
|  |  | 2 | 24.7 | 88.5 | 59.4 |
|  | Input | 1 | 31.6 | 94.2 | 67.4 |
|  |  | 2 | 38.5 | 93.5 | 67.2 |
| FL HSC | H3K4me1 | 1 | 23.1 | 94 | 71.9 |
|  |  | 2 | 26.4 | 97.7 | 72 |
|  | H3K4me3 | 1 | 22.9 | 93.1 | 66.8 |
|  |  | 2 | 22.8 | 93.1 | 66.9 |
|  | H3K27ac | 1 | 30.1 | 89.4 | 67.1 |
|  |  | 2 | 37.6 | 88.6 | 66.3 |
|  | H3K27me3 | 1 | 26.4 | 94 | 63.5 |
|  |  | 2 | 36.3 | 94 | 63.6 |
|  | Input | 1 | 34.4 | 97.8 | 68.9 |
|  |  | 2 | 29.2 | 97.7 | 69.7 |
| Pre-HSC | H3K4me1 | 1 | 32.5 | 96.9 | 72.8 |
|  |  | 2 | 25.1 | 96.9 | 73.7 |
|  | H3K4me3 | 1 | 36.7 | 96.5 | 70.1 |
|  |  | 2 | 32.6 | 95.4 | 70.4 |
|  | H3K27ac | 1 | 28.2 | 96.2 | 67.9 |
|  |  | 2 | 29 | 96 | 67.6 |
|  | H3K27me3 | 1 | 27.9 | 95.6 | 65.2 |
|  |  | 2 | 27.5 | 96 | 65.8 |
|  | Input | 1 | 24.2 | 97.1 | 66.4 |
|  |  | 2 | 37.3 | 97.6 | 66.1 |
| HE | H3K4me1 | 1 | 20.3 | 97.3 | 73.5 |
|  |  | 2 | 35.1 | 97 | 72 |
|  | H3K4me3 | 1 | 49.3 | 95.6 | 69.1 |
|  |  | 2 | 42.6 | 97.6 | 68.1 |
|  | H3K27ac | 1 | 22.7 | 97.7 | 73.7 |
|  |  | 2 | 21.6 | 97.4 | 69.9 |
|  | H3K27me3 | 1 | 31.1 | 94.1 | 65.2 |
|  |  | 2 | 24.7 | 94.5 | 64 |
|  | Input | 1 | 40.2 | 98.3 | 68 |
| Endo | H3K4me1 | 1 | 30.2 | 97.4 | 71.3 |
|  |  | 2 | 23.1 | 95.7 | 71.1 |
|  | H3K4me3 | 1 | 28.8 | 97.3 | 69.1 |
|  |  | 2 | 26.0 | 92.9 | 66.5 |
|  | H3K27ac | 1 | 20.1 | 96.8 | 69.3 |
|  |  | 2 | 21.3 | 96.1 | 69.2 |
|  | H3K27me3 | 1 | 22.9 | 95.9 | 66.7 |
|  |  | 2 | 23.8 | 96.8 | 67.2 |
|  | Input | 1 | 33.3 | 98.2 | 66.8 |
|  |  | 2 | 18.7 | 98.3 | 68.5 |
|  |  | 3 | 22.7 | 98.1 | 67.8 |
