# Supplementary material for: Transcriptional regulatory network controlling the ontogeny of hematopoietic stem cells
Source: Genes Dev. 2020 Jul 1;34(13-14):950–64. doi: 10.1101/gad.338202.120 (PMC7328518; doi:10.1101/gad.338202.120)
Supplement: Supplemental Material [file supp_gad.338202.120_Supplemental_Table_S5.docx]

**Supplemental Table S5. Known mouse hematopoietic enhancers.**

| Enhancer target gene | Enhancer genomic coordinates (mm9) | Reference (Pubmed ID) |
| --- | --- | --- |
| *Erg* | chr16:95660425-95661774 | 21536859 |
| *Fli1* | chr9:32336736-32338015 | 17962413 |
| *Gata2* | chr6:88139710-88141149 | 22996665 |
| *Gata2* | chr6:88152927-88153387 | 17395646 |
| *Gfi1* | chr10:108188068-108188538 | 20516218 |
| *Ly6a* | chr15:74818750-74820500 | 12456959 |
| *Runx1* | chr16:92801501-92802550 | 19321859 |
| *Scl* | chr4:114747481-114749220 | 11731461 |
