# Supplementary material for: Transcriptional regulatory network controlling the ontogeny of hematopoietic stem cells
Source: Genes Dev. 2020 Jul 1;34(13-14):950–64. doi: 10.1101/gad.338202.120 (PMC7328518; doi:10.1101/gad.338202.120)
Supplement: Supplemental Material [file supp_gad.338202.120_Supplemental_Table_S11.docx]

**Supplemental Table S11. Sequences of guide RNAs for CRISPR-Cas9-mediated gene knockout in zebrafish.**

| Target gene | Target site (5’ to 3’) |
| --- | --- |
| *sp3a* | TCGATGGCGCAGGACGCCAC |
| *sp3b* | CTGACTGCTGTCCACGTCCA |
| *maza* | CAGCGCGGCGGTATCCACCG |
| *si:ch211-166g5.4* | TACCGCAGGTCTCGCATGCG |
