# Supplementary material for: Transcriptional regulatory network controlling the ontogeny of hematopoietic stem cells
Source: Genes Dev. 2020 Jul 1;34(13-14):950–64. doi: 10.1101/gad.338202.120 (PMC7328518; doi:10.1101/gad.338202.120)
Supplement: Supplemental Material [file supp_gad.338202.120_Supplemental_Table_S12.docx]

**Supplemental Table S12. CRISPR editing efficiency of *sp3 and maz homologs.*** For each homolog pair (*sp3a* + *sp3b*, *maza* + *si:ch211-166g5.4*), up to 10 individual embryos were assessed for the editing efficiency using the Tracking of Indels by Decomposition (TIDE) assay. Values are mean editing efficiencies of the 10 individual embryos.

| Gene | Genome editing efficiency | | | | | | | | | |
| --- | --- | --- | --- | --- | --- | --- | --- | --- | --- | --- |
|  | #1 | #2 | #3 | #4 | #5 | #6 | #7 | #8 | #9 | #10 |
| *sp3a* | 98.6% | 82.9% | 86% | 71.3% | 88% | 96% | 93.8% | 90% | 93.7% | 90.8% |
| *sp3b* | 86.5% | 95.1% | 92.1% | 96.1% | 86.8% | 91.8% | 94.7% | 97.5% | 48.5% | 65.8% |
| *maza* | 98.6% | 82.9% | 86% | 71.3% | 88% | 96% | 93.8% | 90% | 93.7% | 90.8% |
| *si:ch211-166g5.4* | 86.5% | 95.1% | 92.1% | 96.1% | 86.8% | 91.8% | 94.7% | 97.5% | 48.5% | 65.8% |
