# Supplementary material for: Transcriptional regulatory network controlling the ontogeny of hematopoietic stem cells
Source: Genes Dev. 2020 Jul 1;34(13-14):950–64. doi: 10.1101/gad.338202.120 (PMC7328518; doi:10.1101/gad.338202.120)
Supplement: Supplemental Material [file supp_gad.338202.120_Supplemental_Table_S13.docx]

**Supplemental Table S13. Antibodies used in this study.**

| Antibody | Fluorochrome | Cat. No. | Vendor | Purpose |
| --- | --- | --- | --- | --- |
| CD117 (c-Kit) | APC | 17-1171-83 | eBioscience | FACS |
| CD117 (c-Kit) | PerCP-eFluor 710 | 46-1171-82 | eBioscience | FACS |
| CD11b | Alexa Fluor^®^ 488 | 101217 | Biolegend | FACS |
| CD11c | Alexa Fluor^®^ 488 | 117311 | Biolegend | FACS |
| CD135 | BV421 | 562898 | BD Horizon™ | FACS |
| CD144 (VEC) | PE | 12-1441-82 | eBioscience | FACS |
| CD150 | PE-Cyanine7 | 25-1502-82 | eBioscience | FACS |
| CD31 | PE-Cy7 | 25-0311-82 | eBioscience | FACS |
| CD3ε | Alexa Fluor^®^ 488 | 100321 | Biolegend | FACS |
| CD4 | Alexa Fluor^®^ 488 | 100529 | Biolegend | FACS |
| CD41 | FITC | 133904 | Biolegend | FACS |
| CD41 | eFluor 450 | 48-0411-82 | eBioscience | FACS |
| CD41 | PerCP-eFluor 710 | 46-0411-82 | eBioscience | FACS |
| CD44 | APC-Cy7 | 560568 | BD Pharmingen | FACS |
| CD45 | eFluor 450 | 48-0451-82 | eBioscience | FACS |
| CD45 | PE-Cy7 | 25-0451-81 | eBioscience | FACS |
| CD45R/B220 | Alexa Fluor^®^ 488 | 103225 | Biolegend | FACS |
| CD48 | PerCP/Cyanine5.5 | 103422 | Biolegend | FACS |
| CD8a | Alexa Fluor^®^ 488 | 100723 | Biolegend | FACS |
| ESAM | FITC | 136205 | Biolegend | FACS |
| ESAM | APC | 136207 | Biolegend | FACS |
| Ly-6A/E (Sca-1) | PE | 12-5981-83 | eBioscience | FACS |
| Ly-6G/Ly-6C (Gr-1) | Alexa Fluor^®^ 488 | 108417 | Biolegend | FACS |
| TER-119 | Alexa Fluor^®^ 488 | 116215 | Biolegend | FACS |
| TER-119 | eFluor 450 | 48-5921-82 | eBioscience | FACS |
| H3K4me1 | N/A | ab8895 | Abcam | ChIP-Seq |
| H3K4me3 | N/A | 07-473 | Millipore | ChIP-Seq |
| H3K27me3 | N/A | 07-449 | Millipore | ChIP-Seq |
| H3K27ac | N/A | ab4729 | Abcam | ChIP-Seq |
| Sp3+4 | N/A | LS-C354250 | LSBio | Western blot |
| beta actin | N/A | PA5-72633 | Thermo | Western blot |
| CD117 (c-Kit) | N/A | 14-1171-85 | Thermo Fisher | IF |
| CD31 | N/A | 557355 | BD Biosciences | IF |
| RUNX1 | N/A | Ab92336 | Abcam | IF |
| SOX17 | N/A | Ab224637 | Abcam | IF |
| Goat Anti-Rat IgG | Alexa Fluor^®^ 647 | A-21247 | Molecular Probes | IF |
| Goat Anti-Rat IgG | Alexa Fluor^®^ 555 | A-21434 | Molecular Probes | IF |
| Goat Anti-Rabbit IgG | Alexa Fluor^®^ 488 | A-11034 | Thermo Fisher | IF |
