# Supplementary material for: Transcriptional regulatory network controlling the ontogeny of hematopoietic stem cells
Source: Genes Dev. 2020 Jul 1;34(13-14):950–64. doi: 10.1101/gad.338202.120 (PMC7328518; doi:10.1101/gad.338202.120)
Supplement: Supplemental Material [file supp_gad.338202.120_Supplemental_Table_S14.docx]

**Supplemental Table S14. List of PCR primers.**

| Target region | Sequence (5’ to 3’) | Purpose | |
| --- | --- | --- | --- |
| *sp3a* | TACGGGATGATTCAGGTG  CGAGGACGACACTGAAGA | PCR | |
| *sp3b* | ACAAAGCGAAGGCTCACA  ATACCTTTTCACGCTATGACTGG | PCR | |
| *maza* | CCTGCTTCCCAAAAGTATG  CACTGTTATGTCACAATGCG | PCR | |
| *si:ch211-166g5.4* | TTGGTCCTACCGTGAGAAA  ACACAGTGCTCCTCTCTC | PCR | |
| *sp3a* | TCAGGACAGATACAGTGGCAG  TTGGAGCCGTCTGGAGTTG | RT-qPCR | |
| *sp3b* | GGACAGCAGTCAGAGCGA  GCCGTGAGGTCTGTGGTCT | RT-qPCR | |
| *maza* | GCTGAGCGAGGAGCCCAT  TTACAAATGGAGCAGATGTGAGC | RT-qPCR | |
| *si:ch211-166g5.4* | CGTCAGACAGGTCCATTCTTCAG  GGAACCTTCTCTTCGTGTCTGA | RT-qPCR | |
| *actb1* | GAATCCCAAAGCCAACAGAGAG  AGAGCACAGCCTGGATGG | RT-qPCR | |
| *Scl +*19 enhancer | CCCACCTCACTTTGCCTTC  CGAAGATCACATCCTGTTATTGT | H3K4me1 ChIP-qPCR for all 5 cell types  And H3K27ac ChIP-qPCR for BM HSC | |
| *Actb* promoter | TGGACAAAGACCCAGAGGC  AAAAGCCGTATTAGGTCCATCT | H3K4me3 and H3K27ac ChIP-qPCR for  all 5 cell types |  |
| *Gata5* promoter | GAAGAAAGACGGGCAGACG  TGAACCGACTCAAAAAAGTGG | H3K27me3 ChIP-qPCR for BM HSC,  FL HSC, pre-HSC & HE |  |
| *Gata2* -3 enhancer | GATCTGTGGTGGTAAATGCTG  CCTTCCAGATTATCGCAGC | H3K4me1 and H3K27ac ChIP-qPCR for  FL HSC |  |
| *Cdh5* enhancer | GGGCAAGCAGTCTACCAACA  CAAGGCCAGCCTAAGCAATCT | H3K4me1 and H3K27ac ChIP-qPCR for  Endo |  |
| *Htr2c* promoter | GACTCCTCCCCTCATCCC  GAGAACCAGAGCACCTACCC | H3K27me3 ChIP-qPCR for Endo |  |
| a distal region | AACCTCACACACAACAAGCTG  TGTGATAGGGAGAATGCTTGC | Negative control for all ChIP-qPCR |  |
