# Supplementary material for: Transcriptional regulatory network controlling the ontogeny of hematopoietic stem cells
Source: Genes Dev. 2020 Jul 1;34(13-14):950–64. doi: 10.1101/gad.338202.120 (PMC7328518; doi:10.1101/gad.338202.120)
Supplement: Supplemental Material [file supp_gad.338202.120_Supplemental_Table_S16.docx]

**Supplemental Table S16. Number of cells in each population from *Sp3* scRNA-seq.**

| Population | *Sp3* WT | *Sp3* KO |
| --- | --- | --- |
| Arterial E | 499 | 494 |
| Conflux E | 183 | 142 |
| Pre-HE | 135 | 86 |
| HE | 59 | 40 |
| IAC | 127 | 107 |
